# Search for exotic decays of the Higgs boson to a pair of pseudoscalars in the $\upmu \upmu \text{ b } \text{ b }$ and $\uptau \uptau \text{ b } \text{ b }$ final states

**DOI:** 10.1140/epjc/s10052-024-12727-4

**Published:** 2024-05-14

**Authors:** CMS Collaboration, A. Hayrapetyan, A. Tumasyan, W. Adam, J. W. Andrejkovic, T. Bergauer, S. Chatterjee, K. Damanakis, M. Dragicevic, A. Escalante Del Valle, P. S. Hussain, M. Jeitler, N. Krammer, D. Liko, I. Mikulec, J. Schieck, R. Schöfbeck, D. Schwarz, M. Sonawane, S. Templ, W. Waltenberger, C.-E. Wulz, M. R. Darwish, T. Janssen, P. Van Mechelen, E. S. Bols, J. D’Hondt, S. Dansana, A. De Moor, M. Delcourt, H. El Faham, S. Lowette, I. Makarenko, D. Müller, A. R. Sahasransu, S. Tavernier, M. Tytgat, S. Van Putte, D. Vannerom, B. Clerbaux, G. De Lentdecker, L. Favart, D. Hohov, J. Jaramillo, A. Khalilzadeh, K. Lee, M. Mahdavikhorrami, A. Malara, S. Paredes, L. Pétré, N. Postiau, L. Thomas, M. Vanden Bemden, C. Vander Velde, P. Vanlaer, M. De Coen, D. Dobur, Y. Hong, J. Knolle, L. Lambrecht, G. Mestdach, C. Rendón, A. Samalan, K. Skovpen, N. Van Den Bossche, L. Wezenbeek, A. Benecke, G. Bruno, C. Caputo, C. Delaere, I. S. Donertas, A. Giammanco, K. Jaffel, Sa. Jain, V. Lemaitre, J. Lidrych, P. Mastrapasqua, K. Mondal, T. T. Tran, S. Wertz, G. A. Alves, E. Coelho, C. Hensel, T. Menezes De Oliveira, A. Moraes, P. Rebello Teles, M. Soeiro, W. L. Aldá Júnior, M. Alves Gallo Pereira, M. Barroso Ferreira Filho, H. Brandao Malbouisson, W. Carvalho, J. Chinellato, E. M. Da Costa, G. G. Da Silveira, D. De Jesus Damiao, S. Fonseca De Souza, J. Martins, C. Mora Herrera, K. Mota Amarilo, L. Mundim, H. Nogima, A. Santoro, S. M. Silva Do Amaral, A. Sznajder, M. Thiel, A. Vilela Pereira, C. A. Bernardes, L. Calligaris, T. R. Fernandez Perez Tomei, E. M. Gregores, P. G. Mercadante, S. F. Novaes, B. Orzari, Sandra S. Padula, A. Aleksandrov, G. Antchev, R. Hadjiiska, P. Iaydjiev, M. Misheva, M. Shopova, G. Sultanov, A. Dimitrov, T. Ivanov, L. Litov, B. Pavlov, P. Petkov, A. Petrov, E. Shumka, S. Keshri, S. Thakur, T. Cheng, Q. Guo, T. Javaid, M. Mittal, L. Yuan, G. Bauer, Z. Hu, K. Yi, G. M. Chen, H. S. Chen, M. Chen, F. Iemmi, C. H. Jiang, A. Kapoor, H. Liao, Z.-A. Liu, F. Monti, M. A. Shahzad, R. Sharma, J. N. Song, J. Tao, C. Wang, J. Wang, Z. Wang, H. Zhang, A. Agapitos, Y. Ban, A. Levin, C. Li, Q. Li, X. Lyu, Y. Mao, S. J. Qian, X. Sun, D. Wang, H. Yang, C. Zhou, Z. You, N. Lu, X. Gao, D. Leggat, H. Okawa, Y. Zhang, Z. Lin, C. Lu, M. Xiao, C. Avila, D. A. Barbosa Trujillo, A. Cabrera, C. Florez, J. Fraga, J. A. Reyes Vega, J. Mejia Guisao, F. Ramirez, M. Rodriguez, J. D. Ruiz Alvarez, D. Giljanovic, N. Godinovic, D. Lelas, A. Sculac, M. Kovac, T. Sculac, P. Bargassa, V. Brigljevic, B. K. Chitroda, D. Ferencek, S. Mishra, A. Starodumov, T. Susa, A. Attikis, K. Christoforou, S. Konstantinou, J. Mousa, C. Nicolaou, F. Ptochos, P. A. Razis, H. Rykaczewski, H. Saka, A. Stepennov, M. Finger, M. Finger, A. Kveton, E. Ayala, E. Carrera Jarrin, S. Elgammal, A. Ellithi Kamel, A. Lotfy, M. A. Mahmoud, R. K. Dewanjee, K. Ehataht, M. Kadastik, T. Lange, S. Nandan, C. Nielsen, J. Pata, M. Raidal, L. Tani, C. Veelken, H. Kirschenmann, K. Osterberg, M. Voutilainen, S. Bharthuar, E. Brücken, F. Garcia, J. Havukainen, K. T. S. Kallonen, M. S. Kim, R. Kinnunen, T. Lampén, K. Lassila-Perini, S. Lehti, T. Lindén, M. Lotti, L. Martikainen, M. Myllymäki, M. M. Rantanen, H. Siikonen, E. Tuominen, J. Tuominiemi, P. Luukka, H. Petrow, T. Tuuva, M. Besancon, F. Couderc, M. Dejardin, D. Denegri, J. L. Faure, F. Ferri, S. Ganjour, P. Gras, G. Hamel de Monchenault, V. Lohezic, J. Malcles, J. Rander, A. Rosowsky, M. Ö. Sahin, A. Savoy-Navarro, P. Simkina, M. Titov, C. Baldenegro Barrera, F. Beaudette, A. Buchot Perraguin, P. Busson, A. Cappati, C. Charlot, F. Damas, O. Davignon, A. De Wit, G. Falmagne, B. A. Fontana Santos Alves, S. Ghosh, A. Gilbert, R. Granier de Cassagnac, A. Hakimi, B. Harikrishnan, L. Kalipoliti, G. Liu, J. Motta, M. Nguyen, C. Ochando, L. Portales, R. Salerno, U. Sarkar, J. B. Sauvan, Y. Sirois, A. Tarabini, E. Vernazza, A. Zabi, A. Zghiche, J.-L. Agram, J. Andrea, D. Apparu, D. Bloch, J.-M. Brom, E. C. Chabert, C. Collard, S. Falke, U. Goerlach, C. Grimault, R. Haeberle, A.-C. Le Bihan, M. A. Sessini, P. Van Hove, S. Beauceron, B. Blancon, G. Boudoul, N. Chanon, J. Choi, D. Contardo, P. Depasse, C. Dozen, H. El Mamouni, J. Fay, S. Gascon, M. Gouzevitch, C. Greenberg, G. Grenier, B. Ille, I. B. Laktineh, M. Lethuillier, L. Mirabito, S. Perries, M. Vander Donckt, P. Verdier, J. Xiao, A. Khvedelidze, I. Lomidze, Z. Tsamalaidze, V. Botta, L. Feld, K. Klein, M. Lipinski, D. Meuser, A. Pauls, N. Röwert, M. Teroerde, S. Diekmann, A. Dodonova, N. Eich, D. Eliseev, F. Engelke, M. Erdmann, P. Fackeldey, B. Fischer, T. Hebbeker, K. Hoepfner, F. Ivone, A. Jung, M. Y. Lee, L. Mastrolorenzo, M. Merschmeyer, A. Meyer, S. Mukherjee, D. Noll, A. Novak, F. Nowotny, A. Pozdnyakov, Y. Rath, W. Redjeb, F. Rehm, H. Reithler, V. Sarkisovi, A. Schmidt, S. C. Schuler, A. Sharma, A. Stein, F. Torres Da Silva De Araujo, L. Vigilante, S. Wiedenbeck, S. Zaleski, C. Dziwok, G. Flügge, W. Haj Ahmad, T. Kress, A. Nowack, O. Pooth, A. Stahl, T. Ziemons, A. Zotz, H. Aarup Petersen, M. Aldaya Martin, J. Alimena, S. Amoroso, Y. An, S. Baxter, M. Bayatmakou, H. Becerril Gonzalez, O. Behnke, A. Belvedere, S. Bhattacharya, F. Blekman, K. Borras, D. Brunner, A. Campbell, A. Cardini, C. Cheng, F. Colombina, S. Consuegra Rodríguez, G. Correia Silva, M. De Silva, G. Eckerlin, D. Eckstein, L. I. Estevez Banos, O. Filatov, E. Gallo, A. Geiser, A. Giraldi, G. Greau, V. Guglielmi, M. Guthoff, A. Hinzmann, A. Jafari, L. Jeppe, N. Z. Jomhari, B. Kaech, M. Kasemann, H. Kaveh, C. Kleinwort, R. Kogler, M. Komm, D. Krücker, W. Lange, D. Leyva Pernia, K. Lipka, W. Lohmann, R. Mankel, I.-A. Melzer-Pellmann, M. Mendizabal Morentin, J. Metwally, A. B. Meyer, G. Milella, A. Mussgiller, A. Nürnberg, Y. Otarid, D. Pérez Adán, E. Ranken, A. Raspereza, B. Ribeiro Lopes, J. Rübenach, A. Saggio, M. Scham, S. Schnake, P. Schütze, C. Schwanenberger, D. Selivanova, M. Shchedrolosiev, R. E. Sosa Ricardo, L. P. Sreelatha Pramod, D. Stafford, F. Vazzoler, A. Ventura Barroso, R. Walsh, Q. Wang, Y. Wen, K. Wichmann, L. Wiens, C. Wissing, S. Wuchterl, Y. Yang, A. Zimermmane Castro Santos, A. Albrecht, S. Albrecht, M. Antonello, S. Bein, L. Benato, M. Bonanomi, P. Connor, M. Eich, K. El Morabit, Y. Fischer, A. Fröhlich, C. Garbers, E. Garutti, A. Grohsjean, M. Hajheidari, J. Haller, H. R. Jabusch, G. Kasieczka, P. Keicher, R. Klanner, W. Korcari, T. Kramer, V. Kutzner, F. Labe, J. Lange, A. Lobanov, C. Matthies, A. Mehta, L. Moureaux, M. Mrowietz, A. Nigamova, Y. Nissan, A. Paasch, K. J. Pena Rodriguez, T. Quadfasel, B. Raciti, M. Rieger, D. Savoiu, J. Schindler, P. Schleper, M. Schröder, J. Schwandt, M. Sommerhalder, H. Stadie, G. Steinbrück, A. Tews, M. Wolf, S. Brommer, M. Burkart, E. Butz, T. Chwalek, A. Dierlamm, A. Droll, N. Faltermann, M. Giffels, A. Gottmann, F. Hartmann, R. Hofsaess, M. Horzela, U. Husemann, M. Klute, R. Koppenhöfer, M. Link, A. Lintuluoto, S. Maier, S. Mitra, M. Mormile, Th. Müller, M. Neukum, M. Oh, G. Quast, K. Rabbertz, B. Regnery, N. Shadskiy, I. Shvetsov, H. J. Simonis, N. Trevisani, R. Ulrich, J. van der Linden, R. F. Von Cube, M. Wassmer, S. Wieland, F. Wittig, R. Wolf, S. Wunsch, X. Zuo, G. Anagnostou, P. Assiouras, G. Daskalakis, A. Kyriakis, A. Papadopoulos, A. Stakia, P. Kontaxakis, G. Melachroinos, A. Panagiotou, I. Papavergou, I. Paraskevas, N. Saoulidou, K. Theofilatos, E. Tziaferi, K. Vellidis, I. Zisopoulos, G. Bakas, T. Chatzistavrou, G. Karapostoli, K. Kousouris, I. Papakrivopoulos, E. Siamarkou, G. Tsipolitis, A. Zacharopoulou, K. Adamidis, I. Bestintzanos, I. Evangelou, C. Foudas, P. Gianneios, C. Kamtsikis, P. Katsoulis, P. Kokkas, P. G. Kosmoglou Kioseoglou, N. Manthos, I. Papadopoulos, J. Strologas, M. Bartók, C. Hajdu, D. Horvath, F. Sikler, V. Veszpremi, M. Csanád, K. Farkas, M. M. A. Gadallah, Á. Kadlecsik, P. Major, K. Mandal, G. Pásztor, A. J. Rádl, G. I. Veres, P. Raics, B. Ujvari, G. Zilizi, G. Bencze, S. Czellar, J. Karancsi, J. Molnar, Z. Szillasi, T. Csorgo, F. Nemes, T. Novak, J. Babbar, S. Bansal, S. B. Beri, V. Bhatnagar, G. Chaudhary, S. Chauhan, N. Dhingra, R. Gupta, A. Kaur, A. Kaur, H. Kaur, M. Kaur, S. Kumar, M. Meena, K. Sandeep, T. Sheokand, J. B. Singh, A. Singla, A. Ahmed, A. Bhardwaj, A. Chhetri, B. C. Choudhary, A. Kumar, M. Naimuddin, K. Ranjan, S. Saumya, S. Acharya, S. Baradia, S. Barman, S. Bhattacharya, D. Bhowmik, S. Dutta, S. Dutta, B. Gomber, P. Palit, G. Saha, B. Sahu, S. Sarkar, M. M. Ameen, P. K. Behera, S. C. Behera, S. Chatterjee, P. Jana, P. Kalbhor, J. R. Komaragiri, D. Kumar, L. Panwar, R. Pradhan, P. R. Pujahari, N. R. Saha, A. Sharma, A. K. Sikdar, S. Verma, T. Aziz, I. Das, S. Dugad, M. Kumar, G. B. Mohanty, P. Suryadevara, A. Bala, S. Banerjee, R. M. Chatterjee, M. Guchait, Sh. Jain, S. Karmakar, S. Kumar, G. Majumder, K. Mazumdar, S. Mukherjee, A. Thachayath, S. Bahinipati, A. K. Das, C. Kar, D. Maity, P. Mal, T. Mishra, V. K. Muraleedharan Nair Bindhu, K. Naskar, A. Nayak, P. Sadangi, P. Saha, S. K. Swain, S. Varghese, D. Vats, A. Alpana, S. Dube, B. Kansal, A. Laha, A. Rastogi, S. Sharma, H. Bakhshiansohi, E. Khazaie, M. Zeinali, S. Chenarani, S. M. Etesami, M. Khakzad, M. Mohammadi Najafabadi, M. Grunewald, M. Abbrescia, R. Aly, A. Colaleo, D. Creanza, B. D’Anzi, N. De Filippis, M. De Palma, A. Di Florio, W. Elmetenawee, L. Fiore, G. Iaselli, G. Maggi, M. Maggi, I. Margjeka, V. Mastrapasqua, S. My, S. Nuzzo, A. Pellecchia, A. Pompili, G. Pugliese, R. Radogna, G. Ramirez-Sanchez, D. Ramos, A. Ranieri, L. Silvestris, F. M. Simone, Ü. Sözbilir, A. Stamerra, R. Venditti, P. Verwilligen, A. Zaza, G. Abbiendi, C. Battilana, D. Bonacorsi, L. Borgonovi, R. Campanini, P. Capiluppi, A. Castro, F. R. Cavallo, M. Cuffiani, G. M. Dallavalle, T. Diotalevi, A. Fanfani, D. Fasanella, P. Giacomelli, L. Giommi, C. Grandi, L. Guiducci, S. Lo Meo, L. Lunerti, S. Marcellini, G. Masetti, F. L. Navarria, A. Perrotta, F. Primavera, A. M. Rossi, T. Rovelli, G. P. Siroli, S. Costa, A. Di Mattia, R. Potenza, A. Tricomi, C. Tuve, G. Barbagli, G. Bardelli, B. Camaiani, A. Cassese, R. Ceccarelli, V. Ciulli, C. Civinini, R. D’Alessandro, E. Focardi, T. Kello, G. Latino, P. Lenzi, M. Lizzo, M. Meschini, S. Paoletti, A. Papanastassiou, G. Sguazzoni, L. Viliani, L. Benussi, S. Bianco, S. Meola, D. Piccolo, P. Chatagnon, F. Ferro, E. Robutti, S. Tosi, A. Benaglia, G. Boldrini, F. Brivio, F. Cetorelli, F. De Guio, M. E. Dinardo, P. Dini, S. Gennai, A. Ghezzi, P. Govoni, L. Guzzi, M. T. Lucchini, M. Malberti, S. Malvezzi, A. Massironi, D. Menasce, L. Moroni, M. Paganoni, D. Pedrini, B. S. Pinolini, S. Ragazzi, N. Redaelli, T. Tabarelli de Fatis, D. Zuolo, S. Buontempo, A. Cagnotta, F. Carnevali, N. Cavallo, A. De Iorio, F. Fabozzi, A. O. M. Iorio, L. Lista, P. Paolucci, B. Rossi, C. Sciacca, R. Ardino, P. Azzi, N. Bacchetta, D. Bisello, P. Bortignon, A. Bragagnolo, R. Carlin, P. Checchia, T. Dorigo, U. Gasparini, G. Grosso, L. Layer, E. Lusiani, M. Margoni, A. T. Meneguzzo, M. Michelotto, M. Migliorini, F. Montecassiano, J. Pazzini, P. Ronchese, R. Rossin, F. Simonetto, G. Strong, M. Tosi, A. Triossi, S. Ventura, H. Yarar, P. Zotto, A. Zucchetta, G. Zumerle, S. Abu Zeid, C. Aimè, A. Braghieri, S. Calzaferri, D. Fiorina, P. Montagna, V. Re, C. Riccardi, P. Salvini, I. Vai, P. Vitulo, S. Ajmal, P. Asenov, G. M. Bilei, D. Ciangottini, L. Fanò, M. Magherini, G. Mantovani, V. Mariani, M. Menichelli, F. Moscatelli, A. Piccinelli, M. Presilla, A. Rossi, A. Santocchia, D. Spiga, T. Tedeschi, P. Azzurri, G. Bagliesi, R. Bhattacharya, L. Bianchini, T. Boccali, E. Bossini, D. Bruschini, R. Castaldi, M. A. Ciocci, M. Cipriani, V. D’Amante, R. Dell’Orso, S. Donato, A. Giassi, F. Ligabue, D. Matos Figueiredo, A. Messineo, M. Musich, F. Palla, S. Parolia, A. Rizzi, G. Rolandi, S. Roy Chowdhury, T. Sarkar, A. Scribano, P. Spagnolo, R. Tenchini, G. Tonelli, N. Turini, A. Venturi, P. G. Verdini, P. Barria, M. Campana, F. Cavallari, L. Cunqueiro Mendez, D. Del Re, E. Di Marco, M. Diemoz, F. Errico, E. Longo, P. Meridiani, J. Mijuskovic, G. Organtini, F. Pandolfi, R. Paramatti, C. Quaranta, S. Rahatlou, C. Rovelli, F. Santanastasio, L. Soffi, R. Tramontano, N. Amapane, R. Arcidiacono, S. Argiro, M. Arneodo, N. Bartosik, R. Bellan, A. Bellora, C. Biino, N. Cartiglia, M. Costa, R. Covarelli, N. Demaria, L. Finco, M. Grippo, B. Kiani, F. Legger, F. Luongo, C. Mariotti, S. Maselli, A. Mecca, E. Migliore, M. Monteno, R. Mulargia, M. M. Obertino, G. Ortona, L. Pacher, N. Pastrone, M. Pelliccioni, M. Ruspa, F. Siviero, V. Sola, A. Solano, D. Soldi, A. Staiano, C. Tarricone, M. Tornago, D. Trocino, G. Umoret, E. Vlasov, S. Belforte, V. Candelise, M. Casarsa, F. Cossutti, K. De Leo, G. Della Ricca, S. Dogra, J. Hong, C. Huh, B. Kim, D. H. Kim, J. Kim, H. Lee, S. W. Lee, C. S. Moon, Y. D. Oh, M. S. Ryu, S. Sekmen, Y. C. Yang, G. Bak, P. Gwak, H. Kim, D. H. Moon, E. Asilar, D. Kim, T. J. Kim, J. A. Merlin, J. Park, S. Choi, S. Han, B. Hong, K. Lee, K. S. Lee, S. Lee, J. Park, S. K. Park, J. Yoo, J. Goh, H. S. Kim, Y. Kim, S. Lee, J. Almond, J. H. Bhyun, J. Choi, W. Jun, J. Kim, J. S. Kim, S. Ko, H. Kwon, H. Lee, J. Lee, J. Lee, B. H. Oh, S. B. Oh, H. Seo, U. K. Yang, I. Yoon, W. Jang, D. Y. Kang, Y. Kang, S. Kim, B. Ko, J. S. H. Lee, Y. Lee, I. C. Park, Y. Roh, I. J. Watson, S. Yang, S. Ha, H. D. Yoo, M. Choi, M. R. Kim, H. Lee, Y. Lee, I. Yu, T. Beyrouthy, Y. Maghrbi, K. Dreimanis, A. Gaile, G. Pikurs, A. Potrebko, M. Seidel, V. Veckalns, N. R. Strautnieks, M. Ambrozas, A. Juodagalvis, A. Rinkevicius, G. Tamulaitis, N. Bin Norjoharuddeen, I. Yusuff, Z. Zolkapli, J. F. Benitez, A. Castaneda Hernandez, H. A. Encinas Acosta, L. G. Gallegos Maríñez, M. León Coello, J. A. Murillo Quijada, A. Sehrawat, L. Valencia Palomo, G. Ayala, H. Castilla-Valdez, E. De La Cruz-Burelo, I. Heredia-De La Cruz, R. Lopez-Fernandez, C. A. Mondragon Herrera, A. Sánchez Hernández, C. Oropeza Barrera, M. Ramírez García, I. Bautista, I. Pedraza, H. A. Salazar Ibarguen, C. Uribe Estrada, I. Bubanja, N. Raicevic, P. H. Butler, A. Ahmad, M. I. Asghar, A. Awais, M. I. M. Awan, H. R. Hoorani, W. A. Khan, V. Avati, L. Grzanka, M. Malawski, H. Bialkowska, M. Bluj, B. Boimska, M. Górski, M. Kazana, M. Szleper, P. Zalewski, K. Bunkowski, K. Doroba, A. Kalinowski, M. Konecki, J. Krolikowski, A. Muhammad, K. Pozniak, W. Zabolotny, M. Araujo, D. Bastos, C. Beirão Da Cruz E Silva, A. Boletti, M. Bozzo, P. Faccioli, M. Gallinaro, J. Hollar, N. Leonardo, T. Niknejad, A. Petrilli, M. Pisano, J. Seixas, J. Varela, J. W. Wulff, P. Adzic, P. Milenovic, M. Dordevic, J. Milosevic, V. Rekovic, M. Aguilar-Benitez, J. Alcaraz Maestre, Cristina F. Bedoya, M. Cepeda, M. Cerrada, N. Colino, B. De La Cruz, A. Delgado Peris, D. Fernández Del Val, J. P. Fernández Ramos, J. Flix, M. C. Fouz, O. Gonzalez Lopez, S. Goy Lopez, J. M. Hernandez, M. I. Josa, J. León Holgado, D. Moran, C. M. Morcillo Perez, Á. Navarro Tobar, C. Perez Dengra, A. Pérez-Calero Yzquierdo, J. Puerta Pelayo, I. Redondo, D. D. Redondo Ferrero, L. Romero, S. Sánchez Navas, L. Urda Gómez, J. Vazquez Escobar, C. Willmott, J. F. de Trocóniz, B. Alvarez Gonzalez, J. Cuevas, J. Fernandez Menendez, S. Folgueras, I. Gonzalez Caballero, J. R. González Fernández, E. Palencia Cortezon, C. Ramón Álvarez, V. Rodríguez Bouza, A. Soto Rodríguez, A. Trapote, C. Vico Villalba, P. Vischia, S. Bhowmik, S. Blanco Fernández, J. A. Brochero Cifuentes, I. J. Cabrillo, A. Calderon, J. Duarte Campderros, M. Fernandez, C. Fernandez Madrazo, G. Gomez, C. Lasaosa García, C. Martinez Rivero, P. Martinez Ruiz del Arbol, F. Matorras, P. Matorras Cuevas, E. Navarrete Ramos, J. Piedra Gomez, L. Scodellaro, I. Vila, J. M. Vizan Garcia, M. K. Jayananda, B. Kailasapathy, D. U. J. Sonnadara, D. D. C. Wickramarathna, W. G. D. Dharmaratna, K. Liyanage, N. Perera, N. Wickramage, D. Abbaneo, C. Amendola, E. Auffray, G. Auzinger, J. Baechler, D. Barney, A. Bermúdez Martínez, M. Bianco, B. Bilin, A. A. Bin Anuar, A. Bocci, E. Brondolin, C. Caillol, T. Camporesi, G. Cerminara, N. Chernyavskaya, D. d’Enterria, A. Dabrowski, A. David, A. De Roeck, M. M. Defranchis, M. Deile, M. Dobson, F. Fallavollita, L. Forthomme, G. Franzoni, W. Funk, S. Giani, D. Gigi, K. Gill, F. Glege, L. Gouskos, M. Haranko, J. Hegeman, V. Innocente, T. James, P. Janot, J. Kieseler, S. Laurila, P. Lecoq, E. Leutgeb, C. Lourenço, B. Maier, L. Malgeri, M. Mannelli, A. C. Marini, F. Meijers, S. Mersi, E. Meschi, V. Milosevic, F. Moortgat, M. Mulders, S. Orfanelli, F. Pantaleo, M. Peruzzi, G. Petrucciani, A. Pfeiffer, M. Pierini, D. Piparo, H. Qu, D. Rabady, G. Reales Gutiérrez, M. Rovere, H. Sakulin, S. Scarfi, M. Selvaggi, A. Sharma, K. Shchelina, P. Silva, P. Sphicas, A. G. Stahl Leiton, A. Steen, S. Summers, D. Treille, P. Tropea, A. Tsirou, D. Walter, J. Wanczyk, K. A. Wozniak, P. Zehetner, P. Zejdl, W. D. Zeuner, T. Bevilacqua, L. Caminada, A. Ebrahimi, W. Erdmann, R. Horisberger, Q. Ingram, H. C. Kaestli, D. Kotlinski, C. Lange, M. Missiroli, L. Noehte, T. Rohe, T. K. Aarrestad, K. Androsov, M. Backhaus, A. Calandri, C. Cazzaniga, K. Datta, A. De Cosa, G. Dissertori, M. Dittmar, M. Donegà, F. Eble, M. Galli, K. Gedia, F. Glessgen, C. Grab, D. Hits, W. Lustermann, A.-M. Lyon, R. A. Manzoni, M. Marchegiani, L. Marchese, C. Martin Perez, A. Mascellani, F. Nessi-Tedaldi, F. Pauss, V. Perovic, S. Pigazzini, M. G. Ratti, M. Reichmann, C. Reissel, T. Reitenspiess, B. Ristic, F. Riti, D. Ruini, D. A. Sanz Becerra, R. Seidita, J. Steggemann, D. Valsecchi, R. Wallny, C. Amsler, P. Bärtschi, C. Botta, D. Brzhechko, M. F. Canelli, K. Cormier, R. Del Burgo, J. K. Heikkilä, M. Huwiler, W. Jin, A. Jofrehei, B. Kilminster, S. Leontsinis, S. P. Liechti, A. Macchiolo, P. Meiring, V. M. Mikuni, U. Molinatti, I. Neutelings, A. Reimers, P. Robmann, S. Sanchez Cruz, K. Schweiger, M. Senger, Y. Takahashi, C. Adloff, C. M. Kuo, W. Lin, P. K. Rout, P. C. Tiwari, S. S. Yu, L. Ceard, Y. Chao, K. F. Chen, P. S. Chen, Z. G. Chen, W.-S. Hou, T. H. Hsu, Y. W. Kao, R. Khurana, G. Kole, Y. Y. Li, R.-S. Lu, E. Paganis, A. Psallidas, X. F. Su, J. Thomas-Wilsker, H. y. Wu, E. Yazgan, C. Asawatangtrakuldee, N. Srimanobhas, V. Wachirapusitanand, D. Agyel, F. Boran, Z. S. Demiroglu, F. Dolek, I. Dumanoglu, E. Eskut, Y. Guler, E. Gurpinar Guler, C. Isik, O. Kara, A. Kayis Topaksu, U. Kiminsu, G. Onengut, K. Ozdemir, A. Polatoz, B. Tali, U. G. Tok, S. Turkcapar, E. Uslan, I. S. Zorbakir, M. Yalvac, B. Akgun, I. O. Atakisi, E. Gülmez, M. Kaya, O. Kaya, S. Tekten, A. Cakir, K. Cankocak, Y. Komurcu, S. Sen, O. Aydilek, S. Cerci, V. Epshteyn, B. Hacisahinoglu, I. Hos, B. Isildak, B. Kaynak, S. Ozkorucuklu, O. Potok, H. Sert, C. Simsek, D. Sunar Cerci, C. Zorbilmez, A. Boyaryntsev, B. Grynyov, L. Levchuk, D. Anthony, J. J. Brooke, A. Bundock, F. Bury, E. Clement, D. Cussans, H. Flacher, M. Glowacki, J. Goldstein, H. F. Heath, L. Kreczko, B. Krikler, S. Paramesvaran, S. Seif El Nasr-Storey, V. J. Smith, N. Stylianou, K. Walkingshaw Pass, R. White, A. H. Ball, K. W. Bell, A. Belyaev, C. Brew, R. M. Brown, D. J. A. Cockerill, C. Cooke, K. V. Ellis, K. Harder, S. Harper, M.-L. Holmberg, J. Linacre, K. Manolopoulos, D. M. Newbold, E. Olaiya, D. Petyt, T. Reis, G. Salvi, T. Schuh, C. H. Shepherd-Themistocleous, I. R. Tomalin, T. Williams, R. Bainbridge, P. Bloch, C. E. Brown, O. Buchmuller, V. Cacchio, C. A. Carrillo Montoya, G. S. Chahal, D. Colling, J. S. Dancu, P. Dauncey, G. Davies, J. Davies, M. Della Negra, S. Fayer, G. Fedi, G. Hall, M. H. Hassanshahi, A. Howard, G. Iles, M. Knight, J. Langford, L. Lyons, A.-M. Magnan, S. Malik, A. Martelli, M. Mieskolainen, J. Nash, M. Pesaresi, B. C. Radburn-Smith, A. Richards, A. Rose, C. Seez, R. Shukla, A. Tapper, K. Uchida, G. P. Uttley, L. H. Vage, T. Virdee, M. Vojinovic, N. Wardle, D. Winterbottom, K. Coldham, J. E. Cole, A. Khan, P. Kyberd, I. D. Reid, S. Abdullin, A. Brinkerhoff, B. Caraway, J. Dittmann, K. Hatakeyama, J. Hiltbrand, A. R. Kanuganti, B. McMaster, M. Saunders, S. Sawant, C. Sutantawibul, M. Toms, J. Wilson, R. Bartek, A. Dominguez, C. Huerta Escamilla, A. E. Simsek, R. Uniyal, A. M. Vargas Hernandez, R. Chudasama, S. I. Cooper, S. V. Gleyzer, C. U. Perez, P. Rumerio, E. Usai, C. West, R. Yi, A. Akpinar, A. Albert, D. Arcaro, C. Cosby, Z. Demiragli, C. Erice, E. Fontanesi, D. Gastler, S. Jeon, J. Rohlf, K. Salyer, D. Sperka, D. Spitzbart, I. Suarez, A. Tsatsos, S. Yuan, G. Benelli, X. Coubez, D. Cutts, M. Hadley, U. Heintz, J. M. Hogan, T. Kwon, G. Landsberg, K. T. Lau, D. Li, J. Luo, S. Mondal, M. Narain, N. Pervan, S. Sagir, F. Simpson, M. Stamenkovic, W. Y. Wong, X. Yan, W. Zhang, S. Abbott, J. Bonilla, C. Brainerd, R. Breedon, M. Calderon De La Barca Sanchez, M. Chertok, M. Citron, J. Conway, P. T. Cox, R. Erbacher, F. Jensen, O. Kukral, G. Mocellin, M. Mulhearn, D. Pellett, W. Wei, Y. Yao, F. Zhang, M. Bachtis, R. Cousins, A. Datta, J. Hauser, M. Ignatenko, M. A. Iqbal, T. Lam, E. Manca, W. A. Nash, D. Saltzberg, B. Stone, V. Valuev, R. Clare, M. Gordon, G. Hanson, W. Si, S. Wimpenny, J. G. Branson, S. Cittolin, S. Cooperstein, D. Diaz, J. Duarte, R. Gerosa, L. Giannini, J. Guiang, R. Kansal, V. Krutelyov, R. Lee, J. Letts, M. Masciovecchio, F. Mokhtar, M. Pieri, M. Quinnan, B. V. Sathia Narayanan, V. Sharma, M. Tadel, E. Vourliotis, F. Würthwein, Y. Xiang, A. Yagil, A. Barzdukas, L. Brennan, C. Campagnari, G. Collura, A. Dorsett, J. Incandela, M. Kilpatrick, J. Kim, A. J. Li, P. Masterson, H. Mei, M. Oshiro, J. Richman, U. Sarica, R. Schmitz, F. Setti, J. Sheplock, D. Stuart, S. Wang, A. Bornheim, O. Cerri, A. Latorre, J. M. Lawhorn, J. Mao, H. B. Newman, T. Q. Nguyen, M. Spiropulu, J. R. Vlimant, C. Wang, S. Xie, R. Y. Zhu, J. Alison, S. An, M. B. Andrews, P. Bryant, V. Dutta, T. Ferguson, A. Harilal, C. Liu, T. Mudholkar, S. Murthy, M. Paulini, A. Roberts, A. Sanchez, W. Terrill, J. P. Cumalat, W. T. Ford, A. Hassani, G. Karathanasis, E. MacDonald, N. Manganelli, F. Marini, A. Perloff, C. Savard, N. Schonbeck, K. Stenson, K. A. Ulmer, S. R. Wagner, N. Zipper, J. Alexander, S. Bright-Thonney, X. Chen, D. J. Cranshaw, J. Fan, X. Fan, D. Gadkari, S. Hogan, J. Monroy, J. R. Patterson, J. Reichert, M. Reid, A. Ryd, J. Thom, P. Wittich, R. Zou, M. Albrow, M. Alyari, O. Amram, G. Apollinari, A. Apresyan, L. A. T. Bauerdick, D. Berry, J. Berryhill, P. C. Bhat, K. Burkett, J. N. Butler, A. Canepa, G. B. Cerati, H. W. K. Cheung, F. Chlebana, G. Cummings, J. Dickinson, I. Dutta, V. D. Elvira, Y. Feng, J. Freeman, A. Gandrakota, Z. Gecse, L. Gray, D. Green, A. Grummer, S. Grünendahl, D. Guerrero, O. Gutsche, R. M. Harris, R. Heller, T. C. Herwig, J. Hirschauer, L. Horyn, B. Jayatilaka, S. Jindariani, M. Johnson, U. Joshi, T. Klijnsma, B. Klima, K. H. M. Kwok, S. Lammel, D. Lincoln, R. Lipton, T. Liu, C. Madrid, K. Maeshima, C. Mantilla, D. Mason, P. McBride, P. Merkel, S. Mrenna, S. Nahn, J. Ngadiuba, D. Noonan, V. Papadimitriou, N. Pastika, K. Pedro, C. Pena, F. Ravera, A. Reinsvold Hall, L. Ristori, E. Sexton-Kennedy, N. Smith, A. Soha, L. Spiegel, S. Stoynev, J. Strait, L. Taylor, S. Tkaczyk, N. V. Tran, L. Uplegger, E. W. Vaandering, I. Zoi, C. Aruta, P. Avery, D. Bourilkov, L. Cadamuro, P. Chang, V. Cherepanov, R. D. Field, E. Koenig, M. Kolosova, J. Konigsberg, A. Korytov, K. H. Lo, K. Matchev, N. Menendez, G. Mitselmakher, A. Muthirakalayil Madhu, N. Rawal, D. Rosenzweig, S. Rosenzweig, K. Shi, J. Wang, T. Adams, A. Al Kadhim, A. Askew, N. Bower, R. Habibullah, V. Hagopian, R. Hashmi, R. S. Kim, S. Kim, T. Kolberg, G. Martinez, H. Prosper, P. R. Prova, O. Viazlo, M. Wulansatiti, R. Yohay, J. Zhang, B. Alsufyani, M. M. Baarmand, S. Butalla, T. Elkafrawy, M. Hohlmann, R. Kumar Verma, M. Rahmani, M. R. Adams, C. Bennett, R. Cavanaugh, S. Dittmer, R. Escobar Franco, O. Evdokimov, C. E. Gerber, D. J. Hofman, J. H. Lee, D. S. Lemos, A. H. Merrit, C. Mills, S. Nanda, G. Oh, B. Ozek, D. Pilipovic, T. Roy, S. Rudrabhatla, M. B. Tonjes, N. Varelas, X. Wang, Z. Ye, J. Yoo, M. Alhusseini, D. Blend, K. Dilsiz, L. Emediato, G. Karaman, O. K. Köseyan, J.-P. Merlo, A. Mestvirishvili, J. Nachtman, O. Neogi, H. Ogul, Y. Onel, A. Penzo, C. Snyder, E. Tiras, B. Blumenfeld, L. Corcodilos, J. Davis, A. V. Gritsan, L. Kang, S. Kyriacou, P. Maksimovic, M. Roguljic, J. Roskes, S. Sekhar, M. Swartz, T. Á. Vámi, A. Abreu, L. F. Alcerro Alcerro, J. Anguiano, P. Baringer, A. Bean, Z. Flowers, D. Grove, J. King, G. Krintiras, M. Lazarovits, C. Le Mahieu, C. Lindsey, J. Marquez, N. Minafra, M. Murray, M. Nickel, M. Pitt, S. Popescu, C. Rogan, C. Royon, R. Salvatico, S. Sanders, C. Smith, Q. Wang, G. Wilson, B. Allmond, A. Ivanov, K. Kaadze, A. Kalogeropoulos, D. Kim, Y. Maravin, K. Nam, J. Natoli, D. Roy, G. Sorrentino, F. Rebassoo, D. Wright, E. Adams, A. Baden, O. Baron, A. Belloni, A. Bethani, Y. M. Chen, S. C. Eno, N. J. Hadley, S. Jabeen, R. G. Kellogg, T. Koeth, Y. Lai, S. Lascio, A. C. Mignerey, S. Nabili, C. Palmer, C. Papageorgakis, M. M. Paranjpe, L. Wang, K. Wong, J. Bendavid, W. Busza, I. A. Cali, Y. Chen, M. D’Alfonso, J. Eysermans, C. Freer, G. Gomez-Ceballos, M. Goncharov, P. Harris, D. Hoang, D. Kovalskyi, J. Krupa, L. Lavezzo, Y.-J. Lee, K. Long, C. Mironov, C. Paus, D. Rankin, C. Roland, G. Roland, S. Rothman, Z. Shi, G. S. F. Stephans, J. Wang, Z. Wang, B. Wyslouch, T. J. Yang, B. Crossman, B. M. Joshi, C. Kapsiak, M. Krohn, D. Mahon, J. Mans, B. Marzocchi, S. Pandey, M. Revering, R. Rusack, R. Saradhy, N. Schroeder, N. Strobbe, M. A. Wadud, L. M. Cremaldi, K. Bloom, M. Bryson, D. R. Claes, C. Fangmeier, F. Golf, G. Haza, J. Hossain, C. Joo, I. Kravchenko, I. Reed, J. E. Siado, G. R. Snow, W. Tabb, A. Vagnerini, A. Wightman, F. Yan, D. Yu, A. G. Zecchinelli, G. Agarwal, H. Bandyopadhyay, L. Hay, I. Iashvili, A. Kharchilava, C. McLean, M. Morris, D. Nguyen, J. Pekkanen, S. Rappoccio, H. Rejeb Sfar, A. Williams, G. Alverson, E. Barberis, Y. Haddad, Y. Han, A. Krishna, J. Li, M. Lu, G. Madigan, D. M. Morse, V. Nguyen, T. Orimoto, A. Parker, L. Skinnari, A. Tishelman-Charny, B. Wang, D. Wood, S. Bhattacharya, J. Bueghly, Z. Chen, K. A. Hahn, Y. Liu, Y. Miao, D. G. Monk, M. H. Schmitt, A. Taliercio, M. Velasco, R. Band, R. Bucci, S. Castells, M. Cremonesi, A. Das, R. Goldouzian, M. Hildreth, K. W. Ho, K. Hurtado Anampa, C. Jessop, K. Lannon, J. Lawrence, N. Loukas, L. Lutton, J. Mariano, N. Marinelli, I. Mcalister, T. McCauley, C. Mcgrady, K. Mohrman, C. Moore, Y. Musienko, H. Nelson, M. Osherson, R. Ruchti, A. Townsend, M. Wayne, H. Yockey, M. Zarucki, L. Zygala, A. Basnet, B. Bylsma, M. Carrigan, L. S. Durkin, C. Hill, M. Joyce, A. Lesauvage, M. Nunez Ornelas, K. Wei, B. L. Winer, B. R. Yates, F. M. Addesa, H. Bouchamaoui, P. Das, G. Dezoort, P. Elmer, A. Frankenthal, B. Greenberg, N. Haubrich, S. Higginbotham, G. Kopp, S. Kwan, D. Lange, A. Loeliger, D. Marlow, I. Ojalvo, J. Olsen, D. Stickland, C. Tully, S. Malik, A. S. Bakshi, V. E. Barnes, S. Chandra, R. Chawla, S. Das, A. Gu, L. Gutay, M. Jones, A. W. Jung, D. Kondratyev, A. M. Koshy, M. Liu, G. Negro, N. Neumeister, G. Paspalaki, S. Piperov, A. Purohit, V. Scheurer, J. F. Schulte, M. Stojanovic, J. Thieman, A. K. Virdi, F. Wang, W. Xie, J. Dolen, N. Parashar, A. Pathak, D. Acosta, A. Baty, T. Carnahan, S. Dildick, K. M. Ecklund, P. J. Fernández Manteca, S. Freed, P. Gardner, F. J. M. Geurts, A. Kumar, W. Li, O. Miguel Colin, B. P. Padley, R. Redjimi, J. Rotter, E. Yigitbasi, Y. Zhang, A. Bodek, P. de Barbaro, R. Demina, J. L. Dulemba, C. Fallon, A. Garcia-Bellido, O. Hindrichs, A. Khukhunaishvili, P. Parygin, E. Popova, R. Taus, G. P. Van Onsem, K. Goulianos, B. Chiarito, J. P. Chou, Y. Gershtein, E. Halkiadakis, A. Hart, M. Heindl, D. Jaroslawski, O. Karacheban, I. Laflotte, A. Lath, R. Montalvo, K. Nash, H. Routray, S. Salur, S. Schnetzer, S. Somalwar, R. Stone, S. A. Thayil, S. Thomas, J. Vora, H. Wang, H. Acharya, D. Ally, A. G. Delannoy, S. Fiorendi, T. Holmes, N. Karunarathna, L. Lee, E. Nibigira, S. Spanier, D. Aebi, M. Ahmad, O. Bouhali, M. Dalchenko, R. Eusebi, J. Gilmore, T. Huang, T. Kamon, H. Kim, S. Luo, S. Malhotra, R. Mueller, D. Overton, D. Rathjens, A. Safonov, N. Akchurin, J. Damgov, V. Hegde, A. Hussain, Y. Kazhykarim, K. Lamichhane, S. W. Lee, A. Mankel, T. Mengke, S. Muthumuni, T. Peltola, I. Volobouev, A. Whitbeck, E. Appelt, S. Greene, A. Gurrola, W. Johns, R. Kunnawalkam Elayavalli, A. Melo, F. Romeo, P. Sheldon, S. Tuo, J. Velkovska, J. Viinikainen, B. Cardwell, B. Cox, J. Hakala, R. Hirosky, A. Ledovskoy, A. Li, C. Neu, C. E. Perez Lara, P. E. Karchin, A. Aravind, S. Banerjee, K. Black, T. Bose, S. Dasu, I. De Bruyn, P. Everaerts, C. Galloni, H. He, M. Herndon, A. Herve, C. K. Koraka, A. Lanaro, R. Loveless, J. Madhusudanan Sreekala, A. Mallampalli, A. Mohammadi, S. Mondal, G. Parida, D. Pinna, A. Savin, V. Shang, V. Sharma, W. H. Smith, D. Teague, H. F. Tsoi, W. Vetens, A. Warden, S. Afanasiev, V. Andreev, Yu. Andreev, T. Aushev, M. Azarkin, A. Babaev, A. Belyaev, V. Blinov, E. Boos, V. Borshch, D. Budkouski, V. Bunichev, M. Chadeeva, V. Chekhovsky, R. Chistov, A. Dermenev, T. Dimova, D. Druzhkin, M. Dubinin, L. Dudko, A. Ershov, G. Gavrilov, V. Gavrilov, S. Gninenko, V. Golovtcov, N. Golubev, I. Golutvin, I. Gorbunov, A. Gribushin, Y. Ivanov, V. Kachanov, L. Kardapoltsev, V. Karjavine, A. Karneyeu, V. Kim, M. Kirakosyan, D. Kirpichnikov, M. Kirsanov, V. Klyukhin, O. Kodolova, D. Konstantinov, V. Korenkov, A. Kozyrev, N. Krasnikov, A. Lanev, P. Levchenko, N. Lychkovskaya, V. Makarenko, A. Malakhov, V. Matveev, V. Murzin, A. Nikitenko, S. Obraztsov, V. Oreshkin, V. Palichik, V. Perelygin, M. Perfilov, S. Petrushanko, S. Polikarpov, V. Popov, O. Radchenko, M. Savina, V. Savrin, V. Shalaev, S. Shmatov, S. Shulha, Y. Skovpen, S. Slabospitskii, V. Smirnov, D. Sosnov, V. Sulimov, E. Tcherniaev, A. Terkulov, O. Teryaev, I. Tlisova, A. Toropin, L. Uvarov, A. Uzunian, A. Vorobyev, N. Voytishin, B. S. Yuldashev, A. Zarubin, I. Zhizhin, A. Zhokin

**Affiliations:** 1 Yerevan Physics Institute, Yerevan, Armenia; 2 Institut für Hochenergiephysik, Vienna, Austria; 3 Universiteit Antwerpen, Antwerp, Belgium; 4 Vrije Universiteit Brussel, Brussels, Belgium; 5 Université Libre de Bruxelles, Brussels, Belgium; 6 Ghent University, Ghent, Belgium; 7 Université Catholique de Louvain, Louvain-la-Neuve, Belgium; 8 Centro Brasileiro de Pesquisas Fisicas, Rio de Janeiro, Brazil; 9 Universidade do Estado do Rio de Janeiro, Rio de Janeiro, Brazil; 10 Universidade Estadual Paulista, Universidade Federal do ABC, São Paulo, Brazil; 11 Institute for Nuclear Research and Nuclear Energy, Bulgarian Academy of Sciences, Sofia, Bulgaria; 12 University of Sofia, Sofia, Bulgaria; 13 Instituto De Alta Investigación, Universidad de Tarapacá, Casilla 7 D, Arica, Chile; 14 Beihang University, Beijing, China; 15 Department of Physics, Tsinghua University, Beijing, China; 16 Institute of High Energy Physics, Beijing, China; 17 State Key Laboratory of Nuclear Physics and Technology, Peking University, Beijing, China; 18 Sun Yat-Sen University, Guangzhou, China; 19 University of Science and Technology of China, Hefei, China; 20 Institute of Modern Physics and Key Laboratory of Nuclear Physics and Ion-beam Application (MOE)-Fudan University, Shanghai, China; 21 Zhejiang University, Hangzhou, Zhejiang China; 22 Universidad de Los Andes, Bogotá, Colombia; 23 Universidad de Antioquia, Medellín, Colombia; 24 Faculty of Electrical Engineering, Mechanical Engineering and Naval Architecture, University of Split, Split, Croatia; 25 Faculty of Science, University of Split, Split, Croatia; 26 Institute Rudjer Boskovic, Zagreb, Croatia; 27 University of Cyprus, Nicosia, Cyprus; 28 Charles University, Prague, Czech Republic; 29 Escuela Politecnica Nacional, Quito, Ecuador; 30 Universidad San Francisco de Quito, Quito, Ecuador; 31 Academy of Scientific Research and Technology of the Arab Republic of Egypt, Egyptian Network of High Energy Physics, Cairo, Egypt; 32 Center for High Energy Physics (CHEP-FU), Fayoum University, El-Fayoum, Egypt; 33 National Institute of Chemical Physics and Biophysics, Tallinn, Estonia; 34 Department of Physics, University of Helsinki, Helsinki, Finland; 35 Helsinki Institute of Physics, Helsinki, Finland; 36 Lappeenranta-Lahti University of Technology, Lappeenranta, Finland; 37 IRFU, CEA, Université Paris-Saclay, Gif-sur-Yvette, France; 38 Laboratoire Leprince-Ringuet, CNRS/IN2P3, Ecole Polytechnique, Institut Polytechnique de Paris, Palaiseau, France; 39 Université de Strasbourg, CNRS, IPHC UMR 7178, Strasbourg, France; 40 Institut de Physique des 2 Infinis de Lyon (IP2I), Villeurbanne, France; 41 Georgian Technical University, Tbilisi, Georgia; 42 I. Physikalisches Institut, RWTH Aachen University, Aachen, Germany; 43 III. Physikalisches Institut A, RWTH Aachen University, Aachen, Germany; 44 III. Physikalisches Institut B, RWTH Aachen University, Aachen, Germany; 45 Deutsches Elektronen-Synchrotron, Hamburg, Germany; 46 University of Hamburg, Hamburg, Germany; 47 Karlsruher Institut fuer Technologie, Karlsruhe, Germany; 48 Institute of Nuclear and Particle Physics (INPP), NCSR Demokritos, Agia Paraskevi, Greece; 49 National and Kapodistrian University of Athens, Athens, Greece; 50 National Technical University of Athens, Athens, Greece; 51 University of Ioánnina, Ioannina, Greece; 52 HUN-REN Wigner Research Centre for Physics, Budapest, Hungary; 53 MTA-ELTE Lendület CMS Particle and Nuclear Physics Group, Eötvös Loránd University, Budapest, Hungary; 54 Faculty of Informatics, University of Debrecen, Debrecen, Hungary; 55 Institute of Nuclear Research ATOMKI, Debrecen, Hungary; 56 MATE Institute of Technology, Karoly Robert Campus, Gyongyos, Hungary; 57 Panjab University, Chandigarh, India; 58 University of Delhi, Delhi, India; 59 Saha Institute of Nuclear Physics, HBNI, Kolkata, India; 60 Indian Institute of Technology Madras, Chennai, India; 61 Tata Institute of Fundamental Research-A, Mumbai, India; 62 Tata Institute of Fundamental Research-B, Mumbai, India; 63 National Institute of Science Education and Research, An OCC of Homi Bhabha National Institute, Bhubaneswar, Odisha India; 64 Indian Institute of Science Education and Research (IISER), Pune, India; 65 Isfahan University of Technology, Isfahan, Iran; 66 Institute for Research in Fundamental Sciences (IPM), Tehran, Iran; 67 University College Dublin, Dublin, Ireland; 68 INFN Sezione di Bari, Università di Bari, Politecnico di Bari, Bari, Italy; 69 INFN Sezione di Bologna, Università di Bologna, Bologna, Italy; 70 INFN Sezione di Catania, Università di Catania, Catania, Italy; 71 INFN Sezione di Firenze, Università di Firenze, Florence, Italy; 72 INFN Laboratori Nazionali di Frascati, Frascati, Italy; 73 INFN Sezione di Genova, Università di Genova, Genoa, Italy; 74 INFN Sezione di Milano-Bicocca, Università di Milano-Bicocca, Milan, Italy; 75 INFN Sezione di Napoli, Università di Napoli ‘Federico II’, Naples, Italy; Università della Basilicata, Potenza, Italy; Scuola Superiore Meridionale (SSM), Naples, Italy; 76 INFN Sezione di Padova, Università di Padova, Padua, Italy; Università di Trento, Trento, Italy; 77 INFN Sezione di Pavia, Università di Pavia, Pavia, Italy; 78 INFN Sezione di Perugia, Università di Perugia, Perugia, Italy; 79 INFN Sezione di Pisa, Università di Pisa, Scuola Normale Superiore di Pisa, Pisa, Italy; Università di Siena, Siena, Italy; 80 INFN Sezione di Roma, Sapienza Università di Roma, Rome, Italy; 81 INFN Sezione di Torino, Università di Torino, Turin, Italy; Università del Piemonte Orientale, Novara, Italy; 82 INFN Sezione di Trieste, Università di Trieste, Trieste, Italy; 83 Kyungpook National University, Daegu, Korea; 84 Institute for Universe and Elementary Particles, Chonnam National University, Kwangju, Korea; 85 Hanyang University, Seoul, Korea; 86 Korea University, Seoul, Korea; 87 Department of Physics, Kyung Hee University, Seoul, Korea; 88 Sejong University, Seoul, Korea; 89 Seoul National University, Seoul, Korea; 90 University of Seoul, Seoul, Korea; 91 Department of Physics, Yonsei University, Seoul, Korea; 92 Sungkyunkwan University, Suwon, Korea; 93 College of Engineering and Technology, American University of the Middle East (AUM), Dasman, Kuwait; 94 Riga Technical University, Riga, Latvia; 95 University of Latvia (LU), Riga, Latvia; 96 Vilnius University, Vilnius, Lithuania; 97 National Centre for Particle Physics, Universiti Malaya, Kuala Lumpur, Malaysia; 98 Universidad de Sonora (UNISON), Hermosillo, Mexico; 99 Centro de Investigacion y de Estudios Avanzados del IPN, Mexico City, Mexico; 100 Universidad Iberoamericana, Mexico City, Mexico; 101 Benemerita Universidad Autonoma de Puebla, Puebla, Mexico; 102 University of Montenegro, Podgorica, Montenegro; 103 University of Canterbury, Christchurch, New Zealand; 104 National Centre for Physics, Quaid-I-Azam University, Islamabad, Pakistan; 105 Faculty of Computer Science, Electronics and Telecommunications, AGH University of Krakow, Kraków, Poland; 106 National Centre for Nuclear Research, Swierk, Poland; 107 Institute of Experimental Physics, Faculty of Physics, University of Warsaw, Warsaw, Poland; 108 Warsaw University of Technology, Warsaw, Poland; 109 Laboratório de Instrumentação e Física Experimental de Partículas, Lisbon, Portugal; 110 Faculty of Physics, University of Belgrade, Belgrade, Serbia; 111 VINCA Institute of Nuclear Sciences, University of Belgrade, Belgrade, Serbia; 112 Centro de Investigaciones Energéticas Medioambientales y Tecnológicas (CIEMAT), Madrid, Spain; 113 Universidad Autónoma de Madrid, Madrid, Spain; 114 Instituto Universitario de Ciencias y Tecnologías Espaciales de Asturias (ICTEA), Universidad de Oviedo, Oviedo, Spain; 115 Instituto de Física de Cantabria (IFCA), CSIC-Universidad de Cantabria, Santander, Spain; 116 University of Colombo, Colombo, Sri Lanka; 117 Department of Physics, University of Ruhuna, Matara, Sri Lanka; 118 CERN, European Organization for Nuclear Research, Geneva, Switzerland; 119 Paul Scherrer Institut, Villigen, Switzerland; 120 ETH Zurich-Institute for Particle Physics and Astrophysics (IPA), Zurich, Switzerland; 121 Universität Zürich, Zurich, Switzerland; 122 National Central University, Chung-Li, Taiwan; 123 National Taiwan University (NTU), Taipei, Taiwan; 124 High Energy Physics Research Unit, Department of Physics, Faculty of Science, Chulalongkorn University, Bangkok, Thailand; 125 Physics Department, Science and Art Faculty, Çukurova University, Adana, Turkey; 126 Physics Department, Middle East Technical University, Ankara, Turkey; 127 Bogazici University, Istanbul, Turkey; 128 Istanbul Technical University, Istanbul, Turkey; 129 Istanbul University, Istanbul, Turkey; 130 Institute for Scintillation Materials of National Academy of Science of Ukraine, Kharkiv, Ukraine; 131 National Science Centre, Kharkiv Institute of Physics and Technology, Kharkiv, Ukraine; 132 University of Bristol, Bristol, UK; 133 Rutherford Appleton Laboratory, Didcot, UK; 134 Imperial College, London, UK; 135 Brunel University, Uxbridge, UK; 136 Baylor University, Waco, TX USA; 137 Catholic University of America, Washington, DC USA; 138 The University of Alabama, Tuscaloosa, AL USA; 139 Boston University, Boston, MA USA; 140 Brown University, Providence, RI USA; 141 University of California, Davis, Davis, CA USA; 142 University of California, Los Angeles, CA USA; 143 University of California, Riverside, Riverside, CA USA; 144 University of California, San Diego, La Jolla, CA USA; 145 Department of Physics, University of California, Santa Barbara, Santa Barbara, CA USA; 146 California Institute of Technology, Pasadena, CA USA; 147 Carnegie Mellon University, Pittsburgh, PA USA; 148 University of Colorado Boulder, Boulder, CO USA; 149 Cornell University, Ithaca, NY USA; 150 Fermi National Accelerator Laboratory, Batavia, IL USA; 151 University of Florida, Gainesville, FL USA; 152 Florida State University, Tallahassee, FL USA; 153 Florida Institute of Technology, Melbourne, FL USA; 154 University of Illinois Chicago, Chicago, USA; 155 The University of Iowa, Iowa City, IA USA; 156 Johns Hopkins University, Baltimore, MD USA; 157 The University of Kansas, Lawrence, KS USA; 158 Kansas State University, Manhattan, KS USA; 159 Lawrence Livermore National Laboratory, Livermore, CA USA; 160 University of Maryland, College Park, MD USA; 161 Massachusetts Institute of Technology, Cambridge, MA USA; 162 University of Minnesota, Minneapolis, MN USA; 163 University of Mississippi, Oxford, MS USA; 164 University of Nebraska-Lincoln, Lincoln, NE USA; 165 State University of New York at Buffalo, Buffalo, NY USA; 166 Northeastern University, Boston, MA USA; 167 Northwestern University, Evanston, IL USA; 168 University of Notre Dame, Notre Dame, IN USA; 169 The Ohio State University, Columbus, OH USA; 170 Princeton University, Princeton, NJ USA; 171 University of Puerto Rico, Mayagüez, PR USA; 172 Purdue University, West Lafayette, IN USA; 173 Purdue University Northwest, Hammond, IN USA; 174 Rice University, Houston, TX USA; 175 University of Rochester, Rochester, NY USA; 176 The Rockefeller University, New York, NY USA; 177 Rutgers, The State University of New Jersey, Piscataway, NJ USA; 178 University of Tennessee, Knoxville, TN USA; 179 Texas A &M University, College Station, TX USA; 180 Texas Tech University, Lubbock, TX USA; 181 Vanderbilt University, Nashville, TN USA; 182 University of Virginia, Charlottesville, VA USA; 183 Wayne State University, Detroit, MI USA; 184 University of Wisconsin-Madison, Madison, WI USA; 185 Authors Affiliated with an Institute or an International Laboratory Covered by a Cooperation Agreement with CERN, Geneva, Switzerland; 186 Yerevan State University, Yerevan, Armenia; 187 TU Wien, Vienna, Austria; 188 Institute of Basic and Applied Sciences, Faculty of Engineering, Arab Academy for Science, Technology and Maritime Transport, Alexandria, Egypt; 189 Ghent University, Ghent, Belgium; 190 Universidade Estadual de Campinas, Campinas, Brazil; 191 Federal University of Rio Grande do Sul, Porto Alegre, Brazil; 192 UFMS, Nova Andradina, Brazil; 193 Nanjing Normal University, Nanjing, China; 194 Henan Normal University, Xinxiang, China; 195 The University of Iowa, Iowa City, IA USA; 196 University of Chinese Academy of Sciences, Beijing, China; 197 China Center of Advanced Science and Technology, Beijing, China; 198 University of Chinese Academy of Sciences, Beijing, China; 199 China Spallation Neutron Source, Dongguan, Guangdong China; 200 Université Libre de Bruxelles, Brussels, Belgium; 201 an Institute or an International Laboratory Covered by a Cooperation Agreement with CERN, Geneva, Switzerland; 202 British University in Egypt, Cairo, Egypt; 203 Cairo University, Cairo, Egypt; 204 Birla Institute of Technology, Mesra, Mesra, India; 205 Purdue University, West Lafayette, IN USA; 206 Université de Haute Alsace, Mulhouse, France; 207 Department of Physics, Tsinghua University, Beijing, China; 208 The University of the State of Amazonas, Manaus, Brazil; 209 Erzincan Binali Yildirim University, Erzincan, Turkey; 210 University of Hamburg, Hamburg, Germany; 211 III. Physikalisches Institut A, RWTH Aachen University, Aachen, Germany; 212 Isfahan University of Technology, Isfahan, Iran; 213 Bergische University Wuppertal (BUW), Wuppertal, Germany; 214 Brandenburg University of Technology, Cottbus, Germany; 215 Forschungszentrum Jülich, Jülich, Germany; 216 CERN, European Organization for Nuclear Research, Geneva, Switzerland; 217 Institute of Physics, University of Debrecen, Debrecen, Hungary; 218 Institute of Nuclear Research ATOMKI, Debrecen, Hungary; 219 Universitatea Babes-Bolyai-Facultatea de Fizica, Cluj-Napoca, Romania; 220 Physics Department, Faculty of Science, Assiut University, Asyût, Egypt; 221 HUN-REN Wigner Research Centre for Physics, Budapest, Hungary; 222 Faculty of Informatics, University of Debrecen, Debrecen, Hungary; 223 Punjab Agricultural University, Ludhiana, India; 224 University of Hyderabad, Hyderabad, India; 225 University of Visva-Bharati, Santiniketan, India; 226 Indian Institute of Science (IISc), Bangalore, India; 227 IIT Bhubaneswar, Bhubaneswar, India; 228 Institute of Physics, Bhubaneswar, India; 229 Department of Physics, Isfahan University of Technology, Isfahan, Iran; 230 Sharif University of Technology, Tehran, Iran; 231 Department of Physics, University of Science and Technology of Mazandaran, Behshahr, Iran; 232 Helwan University, Cairo, Egypt; 233 Italian National Agency for New Technologies, Energy and Sustainable Economic Development, Bologna, Italy; 234 Centro Siciliano di Fisica Nucleare e di Struttura Della Materia, Catania, Italy; 235 Università degli Studi Guglielmo Marconi, Rome, Italy; 236 Scuola Superiore Meridionale, Università di Napoli ‘Federico II’, Naples, Italy; 237 Fermi National Accelerator Laboratory, Batavia, IL USA; 238 Università di Napoli ‘Federico II’, Naples, Italy; 239 Ain Shams University, Cairo, Egypt; 240 Consiglio Nazionale delle Ricerche-Istituto Officina dei Materiali, Perugia, Italy; 241 Riga Technical University, Riga, Latvia; 242 Department of Applied Physics, Faculty of Science and Technology, Universiti Kebangsaan Malaysia, Bangi, Malaysia; 243 Consejo Nacional de Ciencia y Tecnología, Mexico City, Mexico; 244 Trincomalee Campus, Eastern University, Sri Lanka, Nilaveli, Sri Lanka; 245 Saegis Campus, Nugegoda, Sri Lanka; 246 INFN Sezione di Pavia, Università di Pavia, Pavia, Italy; 247 National and Kapodistrian University of Athens, Athens, Greece; 248 Ecole Polytechnique Fédérale Lausanne, Lausanne, Switzerland; 249 University of Vienna Faculty of Computer Science, Vienna, Austria; 250 Universität Zürich, Zurich, Switzerland; 251 Stefan Meyer Institute for Subatomic Physics, Vienna, Austria; 252 Laboratoire d’Annecy-le-Vieux de Physique des Particules, IN2P3-CNRS, Annecy-le-Vieux, France; 253 Near East University, Research Center of Experimental Health Science, Mersin, Turkey; 254 Konya Technical University, Konya, Turkey; 255 Izmir Bakircay University, Izmir, Turkey; 256 Adiyaman University, Adiyaman, Turkey; 257 Bozok Universitesi Rektörlügü, Yozgat, Turkey; 258 Marmara University, Istanbul, Turkey; 259 Milli Savunma University, Istanbul, Turkey; 260 Kafkas University, Kars, Turkey; 261 Hacettepe University, Ankara, Turkey; 262 Faculty of Engineering, Istanbul University-Cerrahpasa, Istanbul, Turkey; 263 Yildiz Technical University, Istanbul, Turkey; 264 Vrije Universiteit Brussel, Brussels, Belgium; 265 School of Physics and Astronomy, University of Southampton, Southampton, UK; 266 University of Bristol, Bristol, UK; 267 IPPP Durham University, Durham, UK; 268 Faculty of Science, Monash University, Clayton, Australia; 269 an Institute or an International Laboratory Covered by a Cooperation Agreement with CERN, Geneva, Switzerland; 270 Università di Torino, Turin, Italy; 271 Bethel University, St. Paul, MN USA; 272 Karamanoğlu Mehmetbey University, Karaman, Turkey; 273 California Institute of Technology, Pasadena, CA USA; 274 United States Naval Academy, Annapolis, MD USA; 275 Bingol University, Bingol, Turkey; 276 Georgian Technical University, Tbilisi, Georgia; 277 Sinop University, Sinop, Turkey; 278 Erciyes University, Kayseri, Turkey; 279 Horia Hulubei National Institute of Physics and Nuclear Engineering (IFIN-HH), Bucharest, Romania; 280 Texas A &M University at Qatar, Doha, Qatar; 281 Kyungpook National University, Daegu, Korea; 282 Another Institute or International Laboratory Covered by a Cooperation Agreement with CERN, Geneva, Switzerland; 283 Universiteit Antwerpen, Antwerp, Belgium; 284 Yerevan Physics Institute, Yerevan, Armenia; 285 Northeastern University, Boston, MA USA; 286 Imperial College, London, UK; 287 Institute of Nuclear Physics of the Uzbekistan Academy of Sciences, Tashkent, Uzbekistan; 288 CERN, 1211 Geneva 23, Switzerland

## Abstract

A search for exotic decays of the Higgs boson ($\text {H}$) with a mass of 125$\,\text {Ge}\hspace{-.08em}\text {V}$ to a pair of light pseudoscalars $\text {a}_{1}$ is performed in final states where one pseudoscalar decays to two ${\textrm{b}}$ quarks and the other to a pair of muons or $\tau$ leptons. A data sample of proton–proton collisions at $\sqrt{s}=13\,\text {Te}\hspace{-.08em}\text {V}$ corresponding to an integrated luminosity of 138$\,\text {fb}^{-1}$ recorded with the CMS detector is analyzed. No statistically significant excess is observed over the standard model backgrounds. Upper limits are set at 95% confidence level ($\text {CL}$) on the Higgs boson branching fraction to $\upmu \upmu \text{ b } \text{ b }$ and to $\uptau \uptau \text{ b } \text{ b },$ via a pair of $\text {a}_{1}$s. The limits depend on the pseudoscalar mass $m_{\text {a}_{1}}$ and are observed to be in the range (0.17–3.3) $\times 10^{-4}$ and (1.7–7.7) $\times 10^{-2}$ in the $\upmu \upmu \text{ b } \text{ b }$ and $\uptau \uptau \text{ b } \text{ b }$ final states, respectively. In the framework of models with two Higgs doublets and a complex scalar singlet (2HDM+S), the results of the two final states are combined to determine upper limits on the branching fraction ${\mathcal {B}}(\text {H} \rightarrow \text {a}_{1} \text {a}_{1} \rightarrow \ell \ell \text{ b } \text{ b})$ at 95% $\text {CL}$, with $\ell$ being a muon or a $\uptau$ lepton. For different types of 2HDM+S, upper bounds on the branching fraction ${\mathcal {B}}(\text {H} \rightarrow \text {a}_{1} \text {a}_{1} )$ are extracted from the combination of the two channels. In most of the Type II 2HDM+S parameter space, ${\mathcal {B}}(\text {H} \rightarrow \text {a}_{1} \text {a}_{1} )$ values above 0.23 are excluded at 95% $\text {CL}$ for $m_{\text {a}_{1}}$ values between 15 and 60$\,\text {Ge}\hspace{-.08em}\text {V}$.

## Introduction

The discovery of the Higgs boson ($\text {H}$) by the ATLAS and CMS experiments at the CERN LHC [1–3] strengthened the case for the standard model (SM), which states that the electroweak (EW) symmetry is broken by a complex scalar field [4–9]. However, the SM is not a complete theory as it cannot account for a number of experimental observations. For example, the origin of neutrino mass and dark matter remains unexplained in the SM. Several beyond the SM (BSM) theories address these observations while identifying the 125$\,\text {Ge}\hspace{-.08em}\text {V}$ resonance as part of an extended group of scalar particles. The Two-Higgs-Doublet Models (2HDMs) [10–12] predict five physical scalar and pseudoscalar particles and allow different couplings of each scalar to SM fermions. The two real scalar singlet extension [13, 14] of the SM results in three neutral scalar bosons. A broad class of 2HDMs extended with an additional complex scalar singlet (2HDM+S) contains seven physical scalar and pseudoscalar particles [11]. In all these models, one of the scalars is identified as the discovered Higgs boson with a mass of 125$\,\text {Ge}\hspace{-.08em}\text {V}$.

Recent measurements of the Higgs boson’s couplings at the LHC do not rule out exotic decays of the Higgs boson to BSM particles. The ATLAS and CMS experiments put, respectively, 12 and 16% upper bounds on the branching fraction of the Higgs boson to undetected particles at 95% confidence level (CL) using data collected in 2016–2018 (Run 2) [15, 16]. Given these bounds, it is crucial to examine the data for direct evidence of new particles coupling to the Higgs boson, in particular, to test possible extensions of the SM.

The exotic decay channels may include the Higgs boson decaying to a pair of light pseudoscalar particles that subsequently decay to pairs of SM particles. This signal can be experimentally discriminated from SM Higgs boson decays. These decays arise naturally in the phenomenology of 2HDM+S, which is described here in more detail. The 2HDM+S couplings are such that a fermion can couple to only one of the scalar doublets to avoid flavor changing neutral currents at tree level. Under this condition, four types of 2HDM+S models are possible [11, 17]. While the SM-like couplings of the Higgs boson to fermions and gauge bosons can be preserved, the singlet state of the 2HDM+S can also serve as a dark matter candidate that couples to the Higgs boson [18, 19]. In 2HDM+S scenarios of Type I, the second doublet, $\phi _2,$ can couple to any fermion whereas the first doublet, $\phi _1,$ cannot couple to fermions. In Type II models, $\phi _1$ couples to down-type quarks and charged leptons while $\phi _2$ couples to up-type quarks. This model is close to the next-to-minimal supersymmetric SM (NMSSM), which is a special case of 2HDM+S and provides a solution to the so-called $\mu$-problem [20, 21]. The NMSSM particle spectrum contains two pseudoscalars, $\text {a}_{1}$ and ${\textrm{a}}_2,$ the lighter $\text {a}_{1}$ can have a mass smaller than the Higgs boson to allow $\text {H} \rightarrow \text {a}_{1} \text {a}_{1}$ decays. Another valid extension has quarks coupling to $\phi _2$ and charged leptons coupling to $\phi _1,$ referred to as the Type III or “lepton-specific” model. Finally, in the Type IV or “flipped” model, $\phi _2$ couples to up-type quarks and charged leptons while $\phi _1$ couples to down-type quarks [11, 17].

The branching fraction, ${\mathcal {B}},$ of $\text {a}_{1} \text {a}_{1} \rightarrow$ SM particles depends on the type of 2HDM+S model, the mass of the pseudoscalar, $m_{\text {a}_{1}}$, and the ratio of the vacuum expectation values of the two doublets, $\tan \beta$. The decay width of $\text {a}_{1}$ to fermion pairs depends, in addition, on the mass of the decay products. In Type II 2HDM+S models, ${\mathcal {B}}(\text {a}_{1} \text {a}_{1} \rightarrow \uptau \uptau {\text{ b }}{\text{ b }})$ is slightly above 10%, while it can reach up to $\sim$50% in Type III models. The large predicted branching fraction makes this channel particularly attractive. The decay of $\text {a}_{1}$ pairs to $\upmu \upmu \text{ b } \text{ b }$ has a much smaller branching fraction. In Type III models, for $\tan \beta =2,$ ${\mathcal {B}}(\text {a}_{1} \text {a}_{1} \rightarrow \upmu \upmu \text{ b } \text{ b } )$ is predicted to be about 0.2%. Despite the small branching fraction, this channel can provide competitive results given the high performance of the muon reconstruction and the excellent dimuon mass resolution in CMS. The possibility of the Higgs boson decaying into a pair of $\text {a}_{1}$s is studied in this paper for both $\uptau \uptau {\text{ b }}{\text{ b }}$ and $\upmu \upmu \text{ b } \text{ b }$ decay modes. The gluon-gluon fusion production mechanism (${\text{ g } \text{ g }}$F) constitutes the dominant Higgs bosons production process, with a cross section of $\sigma _\textrm{ggF}^{13 \,\text {Te}\hspace{-.08em}\text {V}}=48.58\pm 1.56\,\text {pb}$ [22] at next-to-next-to-next-to-leading order $({\textrm{N}}^{3}{\textrm{LO}})$ accuracy in perturbative quantum chromodynamics (QCD) and next-to-leading order (NLO) in EW corrections. The contribution of the Higgs boson production through vector boson fusion (VBF) is also taken into account with a cross section of $\sigma _{\text{ q } \text{ q } \text {H} }^{13 \,\text {Te}\hspace{-.08em}\text {V}}=3.72\pm 0.08\,\text {pb}$ [22], which includes NLO QCD and EW corrections.

Similar searches have been performed at the LHC. The latest analysis by the ATLAS Collaboration [23] has placed a strong bound of ${\mathcal {B}}(\text {H} \rightarrow \text {a}_{1} \text {a}_{1} \rightarrow \upmu \upmu \text{ b } \text{ b } )< (0.2$–$4)\times 10^{-4}$ in the range $16<m_{\text {a}_{1}} <62\,\text {Ge}\hspace{-.08em}\text {V},$ using the LHC Run 2 data at $\sqrt{s}=13\,\text {Te}\hspace{-.08em}\text {V},$ extending its prior analysis with a partial data sample [24]. The existing CMS search at this center-of-mass energy [25] is based on a data sample corresponding to an integrated luminosity of $36\,{\,\text {fb}^{-1}}$ and results in an upper limit on ${\mathcal {B}}(\text {H} \rightarrow \text {a}_{1} \text {a}_{1} \rightarrow \upmu \upmu \text{ b } \text{ b } )$ of (1–$7)\times 10^{-4},$ considering $m_{\text {a}_{1}}$ between 20 and 62.5$\,\text {Ge}\hspace{-.08em}\text {V}$. At 8$\,\text {Te}\hspace{-.08em}\text {V}$, the CMS experiment has provided an upper bound of ${\mathcal {B}}(\text {H} \rightarrow \text {a}_{1} \text {a}_{1} \rightarrow \upmu \upmu \text{ b } \text{ b } )< (2$–$8)\times 10^{-4}$ [26]. In the $\uptau \uptau {\text{ b }}{\text{ b }}$ final state, an upper limit of ${\mathcal {B}}(\text {H} \rightarrow \text {a}_{1} \text {a}_{1} \rightarrow \uptau \uptau {\text{ b }}{\text{ b }})<(3$–$12)\times 10^{-2}$ was reported by CMS using a 36$\,\text {fb}^{-1}$ dataset at $\sqrt{s}=13\,\text {Te}\hspace{-.08em}\text {V},$ where $m_{\text {a}_{1}}$ ranged between 15 and 60$\,\text {Ge}\hspace{-.08em}\text {V}$  [27]. The analysis examined both leptonically and hadronically decaying $\uptau$ leptons, the latter denoted by $\uptau _\textrm{h}$.

This paper reports an extension of CMS searches [25, 27] with the proton–proton $(\text{ p } \text{ p})$ collision data collected in Run 2, corresponding to an integrated luminosity of $138{\,\text {fb}^{-1}}$ at 13 $\,\text {Te}\hspace{-.08em}\text {V}$. Improved techniques in these analyses bring higher sensitivity to the allowed branching fractions. In the $\upmu \upmu \text{ b } \text{ b }$ final state, in particular, a more in-depth study of the signal achieves a greater gain in sensitivity than that offered by the additional LHC data alone. This channel looks for a bump over the dimuon mass spectrum after a cut-based event selection. A neural network approach to optimize the signal region (SR) selection provides better sensitivity to the signal processes in the $\uptau \uptau {\text{ b }}{\text{ b }}$ channel. The results in the two final states are combined, and interpretations are provided in different types of 2HDM+S models.

The paper is organized as follows: Sects. 2 and 3 discuss the CMS detector and the simulated data samples used in these analyses. The event reconstruction and event selection procedures are presented in Sects. 4 and 5, respectively. The background prediction methods are described in Sect. 6. Section 7 presents the signal extraction methods, and the discussion of the systematic uncertainties can be found in Sect. 8. Results and interpretations are detailed in Sect. 9 and a summary is presented in Sect. 10. Tabulated results of this analysis are provided in the HEPData record [28].

## The CMS experiment

The central feature of the CMS apparatus is a superconducting solenoid of 6$\,\text {m}$ internal diameter, providing a magnetic field of 3.8$\,\text {T}$. Within the solenoid volume are a silicon pixel and strip tracker, a lead tungstate crystal electromagnetic calorimeter (ECAL), and a brass and scintillator hadron calorimeter (HCAL), each composed of a barrel and two endcap sections. Forward calorimeters extend the pseudorapidity ($\eta$) coverage provided by the barrel and endcap detectors. Muons are detected in gas-ionization chambers embedded in the steel flux-return yoke outside the solenoid. The collision data are recorded with the help of Level-1 (L1) trigger, high-level trigger (HLT), and data acquisition systems ensuring high efficiency in selecting interesting physics events [29]. A more detailed description of the CMS detector, along with a definition of the coordinate system used and the relevant kinematic variables, can be found in Ref. [30].

## Simulated event samples

Simulated samples are used to design and optimize the analysis strategy and, where needed, to estimate background contributions. A number of Monte Carlo (MC) event generators are used to produce events using either leading order (LO) or NLO matrix element calculations. In all cases, parton showering and fragmentation are implemented using pythia (version 8.212) [31]. The description of parton distribution functions (PDFs) relies on the NNPDF3.1 set [32]. Jets produced at the matrix element level are matched with those generated by pythia using the MLM [33, 34] method for LO samples. The FxFx matching [35] is implemented in the case of NLO samples generated with MadGraph 5_amc@nlo (version 2.2.2 for the simulation of the 2016 data and 2.4.2 for 2017–2018) [36]. For the underlying event description, the CUETP8M1 [37] tune was used for MC samples simulating the 2016 data, while for those simulating the 2017–2018 data, the CP5 [38] tune was employed. The Geant4  [39, 40] package has been used for the detector simulation. To model the effect of additional collisions within the same or adjacent bunch crossings (pileup), minimum bias interactions are simulated and superimposed on the hard-scattering events. Simulated events are then reweighted to reproduce the pileup distribution in data.

The $\text {H} \rightarrow \text {a}_{1} \text {a}_{1} \rightarrow \upmu \upmu \text{ b } \text{ b }$ signal events are generated with the NMSSMHET model [17] using MadGraph 5_amc@nlo (version 2.6.5) at LO [34]. Both ${\text{ g } \text{ g }}$F and VBF Higgs boson production mechanisms are considered, within the $\text {a}_{1}$ mass range of 15–60$\,\text {Ge}\hspace{-.08em}\text {V}$. While the ${\text{ g } \text{ g }}$F samples are generated with 5$\,\text {Ge}\hspace{-.08em}\text {V}$ steps in $m_{\text {a}_{1}},$ the VBF samples are generated only for $m_{\text {a}_{1}}$ of 20, 40, and 60$\,\text {Ge}\hspace{-.08em}\text {V}$. Interpolation methods are used to estimate the signal yield and the shape of the dimuon resonance for all mass hypotheses. Similar settings are used to produce $\text {H} \rightarrow \text {a}_{1} \text {a}_{1} \rightarrow \uptau \uptau {\text{ b }}{\text{ b }}$ signal events at 11 $\text {a}_{1}$ masses between 12 and 60$\,\text {Ge}\hspace{-.08em}\text {V}$, for both ${\text{ g } \text{ g }}$F and VBF Higgs boson production modes.

The major backgrounds for the analyses are the Drell–Yan (DY) process (${\text{ Z }}/\gamma ^*$+jets), the production of a top quark–antiquark pair with additional jets (denoted t t+jets), single top quark production, and massive vector boson pair production (Diboson). In the $\upmu \upmu \text{ b } \text{ b }$ channel, the background estimation is performed using methods fully based on control samples in data with no reference to simulation. Simulated background samples are, however, used to optimize the signal selection criteria. In the $\uptau \uptau {\text{ b }}{\text{ b }}$ channel, only the backgrounds from DY production with $\text{ Z } \rightarrow \uptau \uptau ,$ QCD multijet events in the $\text{ e } \hspace{-.04em}\upmu$ final state, and events with jets misidentified as $\uptau _\textrm{h}$ candidates are estimated using control samples in data.

The DY process in the dilepton final state is modeled using MadGraph 5_amc@nlo. Based on the dilepton invariant mass ($m_{\ell \ell }$) threshold at the generator level, two DY samples are considered, one with $m_{\ell \ell } >50\,\text {Ge}\hspace{-.08em}\text {V}$ and the other with $10<m_{\ell \ell } <50\,\text {Ge}\hspace{-.08em}\text {V}.$ The high-mass DY samples are produced at (N)LO, with up to four (two) additional partons at the matrix element level. For the low mass, samples are primarily produced at LO with additional partons, similar to those of high mass, while NLO and LO samples inclusive in number of jets are also utilized. In the $\upmu \upmu \text{ b } \text{ b }$ analysis, the NLO samples at high mass are employed, and at low mass, NLO QCD K-factors are applied to the LO cross section. An uncertainty of 30% is considered on these K-factor corrections, as they are extracted from NLO low-mass samples with limited number of events. The accuracy of the DY sample is found to be sufficient for optimization purposes, which is the only use of the simulated backgrounds in the $\upmu \upmu \text{ b } \text{ b }$ channel. The $\uptau \uptau {\text{ b }}{\text{ b }}$ analysis makes use of LO DY samples in the entire mass range. The cross sections are normalized to next-to-NLO (NNLO) in QCD using K-factors [41]. In addition, the $\text{ Z }$ boson $p_{\textrm{T}}$ distribution is corrected by reweighting simulated events to data in bins of $m_{\ell \ell }$ and the $p_T$ of the dilepton system.

The powheg box v2.0 framework [42–45] event generator is used to produce t t+jets and single top events at NLO. The simulated t t+jets events are reweighted to match the top quark $p_{\textrm{T}}$ distribution at NNLO QCD and NLO EW [46] precision. Diboson and $\text{ W } \hspace{-.04em}$+jets events are generated by MadGraph 5_amc@nlo. Similar to the high-mass DY sample, $\text{ W } \hspace{-.04em}$+jets events are simulated with up to four additional partons at the matrix element level for all years. The t t+jets, DY, and $\text{ W } \hspace{-.04em}$+jets samples are normalized to cross section values accurate to NNLO in QCD [47–55]. All SM backgrounds containing the Higgs boson are generated using powheg v2.0 at NLO [56–60].

## Object reconstruction

The $\upmu \upmu \text{ b } \text{ b }$ and $\uptau \uptau {\text{ b }}{\text{ b }}$ analyses together reconstruct a diverse set of final-state particles for a $\text {H} \rightarrow \text {a}_{1} \text {a}_{1}$ signal. The $\upmu \upmu \text{ b } \text{ b }$ analysis relies on the presence of two prompt muons. In the $\uptau \uptau {\text{ b }}{\text{ b }}$ channel, on the other hand, final states with at least one $\uptau$ lepton decaying to an electron or muon, i.e., $\text{ e } \hspace{-.04em}\upmu$, $\text{ e } \hspace{-.04em}\uptau _\textrm{h}$, and $\upmu \hspace{-.04em}\uptau _\textrm{h}$, are considered. The $\uptau$ lepton decays resulting in same-flavor leptons, or in two $\uptau _\textrm{h}$ candidates, are not included as they bring negligible sensitivity to the analysis. The signal acceptance of $\uptau _\textrm{h} \uptau _\textrm{h}$ is very low due to high trigger thresholds, whereas $\text{ e } \text{ e }$ and $\upmu \upmu$ final states suffer from low branching fractions.

The particle-flow (PF) algorithm [61] is used to reconstruct and identify each individual particle (PF candidate) in the event, with an optimized combination of information from the various elements of the CMS detector. The energy of photons is measured in ECAL. The energy of electrons is determined from a combination of the electron momentum at the primary interaction vertex as measured by the tracker, the energy of the corresponding ECAL cluster, and the energy sum of all bremsstrahlung photons spatially compatible with originating from the electron track. The energy of muons is obtained from the curvature of the corresponding track. The energy of charged hadrons is evaluated via a combination of their momentum measured in the tracker and the matching of the ECAL and HCAL energy deposits, corrected for the response function of the calorimeters to hadronic showers. Finally, the energy of neutral hadrons is obtained from the corresponding corrected ECAL and HCAL energies.

The primary vertex (PV) is taken to be the vertex corresponding to the hardest scattering in the event, identified using the tracking information alone, as described in Section 9.4.1 of Ref. [62].

Muons can be produced directly in $\text {a}_{1}$ decays in the $\upmu \upmu \text{ b } \text{ b }$ final state, or from decays of the $\uptau$ leptons in the $\uptau \uptau {\text{ b }}{\text{ b }}$ channel. In both analyses, muons must lie within $|\eta | < 2.4.$ The $p_{\textrm{T}}$ threshold for muons in the $\uptau \uptau {\text{ b }}{\text{ b }}$ analysis depends on the trigger selection, see Sect. 5 and Table 1. In the $\upmu \hspace{-.04em}\uptau _\textrm{h}$ final state, it is required to be 1$\,\text {Ge}\hspace{-.08em}\text {V}$ greater than the HLT muon $p_{\textrm{T}}$ threshold in order to be in a region where the efficiency of the respective trigger is independent of $p_{\textrm{T}}$. The muon $p_{\textrm{T}}$ requirement in the $\text{ e } \hspace{-.04em}\upmu$ final state, selected with an $\text{ e } \hspace{-.04em}\upmu$ trigger, is 24 (13)$\,\text {Ge}\hspace{-.08em}\text {V}$ when the muon is the leading (subleading) lepton in the pair. In the $\upmu \upmu \text{ b } \text{ b }$ analysis, the leading (subleading) muon $p_{\textrm{T}}$ must exceed 17 (15)$\,\text {Ge}\hspace{-.08em}\text {V}$. The two muons are required to have an opposite electric charge and to be separated by a minimum $\varDelta R \equiv \sqrt{(\varDelta \eta )^{2} + (\varDelta \phi )^{2}} = 0.4,$ where $\phi$ is the azimuthal angle of the particle’s momentum in the plane perpendicular to the beam line. In cases where more than two muons satisfy these criteria, the pair with the highest $p_{\textrm{T}}$ are considered.

In order to suppress contributions from nonprompt decays of hadrons and from their shower penetration in the muon detectors, selected muons must pass dedicated identification requirements. The so-called tight identification [63] is used in the $\upmu \upmu \text{ b } \text{ b }$ analysis with an efficiency varying between 95 and 99%, depending on $\eta ,$ where the data and simulation agree within 1–3%. Looser requirements for muons, known as medium identification criteria [63], are employed in the $\uptau \uptau {\text{ b }}{\text{ b }}$ analysis, with an overall efficiency of 99.5% for simulated $\text{ W }$ and $\text{ Z }$ events.

The lepton isolation variable $I_\text {rel}$ is calculated by summing the transverse energy deposited by other particles in a cone of size $\varDelta R = 0.4$ (0.3) around the muon (electron) and dividing by the lepton $p_{\textrm{T}}$. The contribution of charged particles from pileup is suppressed by requiring the charged particles to be associated with the PV. An average pileup energy is subtracted from the total energy of neutral particles and photons within the isolation cone, since vertex association is not known in this case. Muons are required to pass $I_\text {rel}<0.15$ in the $\uptau \uptau {\text{ b }}{\text{ b }}$ analysis.

In the $\upmu \upmu \text{ b } \text{ b }$ analysis, a looser requirement of $I_\text {rel}<0.25$ is imposed, which results in about 99% efficiency for muons with $p_{\textrm{T}} >20\,\text {Ge}\hspace{-.08em}\text {V},$ independent of $\eta$ [63]. Electrons from $\uptau$ lepton decays are selected within $|\eta |<2.4$ with different $p_{\textrm{T}}$ thresholds, according to the $\uptau \uptau {\text{ b }}{\text{ b }}$ final state. In the $\text{ e } \hspace{-.04em}\upmu$ channel, the threshold is 24$\,\text {Ge}\hspace{-.08em}\text {V}$ if the electron is the leading lepton. Otherwise, it is reduced to 13$\,\text {Ge}\hspace{-.08em}\text {V}$. In the $\text{ e } \hspace{-.04em}\uptau _\textrm{h}$ channel, the electron $p_{\textrm{T}}$ must be more than 1$\,\text {Ge}\hspace{-.08em}\text {V}$ beyond the HLT threshold. A multivariate analysis (MVA) discriminant is used to identify electrons. The MVA exploits several properties of the electron candidate, including energy deposits in the ECAL, the quality of the associated track, and the shower shape in the calorimeters [64]. The chosen MVA working point has a 90% efficiency to correctly identify an electron. Identified electrons are further required to be isolated, fulfilling $I_\text {rel}<0.10.$ In the $\text{ e } \hspace{-.04em}\upmu$ channel of the $\uptau \uptau {\text{ b }}{\text{ b }}$ analysis, the electron must be separated from the muon by $\varDelta R \ge 0.3$ and have an opposite electric charge. For both electrons and muons, correction factors for the reconstruction and identification efficiencies are obtained from data and applied to simulation.

**Table 1:** The electron, muon, and $\uptau _\textrm{h}$ $p_{\textrm{T}}$ thresholds in$\,\text {Ge}\hspace{-.08em}\text {V}$ at trigger level for the $\upmu \upmu \text{ b } \text{ b }$ and $\uptau \uptau {\text{ b }}{\text{ b }}$ channels

Year	Single/dilepton trigger $p_{\textrm{T}}$	$\upmu \upmu \text{ b } \text{ b }$	$\uptau \uptau {\text{ b }}{\text{ b }}$					
			$\text{ e } \hspace{-.04em}\upmu$		$\text{ e } \hspace{-.04em}\uptau _\textrm{h}$		$\upmu \hspace{-.04em}\uptau _\textrm{h}$	
		$\mu$	$\text{ e }$	$\mu$	$\text{ e }$	$\uptau _\textrm{h}$	$\mu$	$\uptau _\textrm{h}$
2016	Single lepton	24	–	–	25	–	22	–
	$p_{\textrm{T}}$-leading lepton	17	23	23	–	–	–	20
	$p_{\textrm{T}}$-subleading lepton	8	12	8			19	–
2017	Single lepton	24	–	–	27, 32	–	24, 27	–
	$p_{\textrm{T}}$-leading lepton	17	23	23	–	30	–	27
	$p_{\textrm{T}}$-subleading lepton	8	12	8	24	–	20	–
2018	Single lepton	24	–	–	32, 35	–	24, 27	–
	$p_{\textrm{T}}$-leading lepton	17	23	23	–	30	–	27
	$p_T$-subleading lepton	8	12	8	24	–	20	–

Jets are reconstructed by clustering the charged and neutral PF candidates using the anti-$k_{\textrm{T}}$ algorithm [65, 66] with a distance parameter of 0.4, up to $|\eta | < 4.7$ for tagging VBF events. The reconstructed jet energy is corrected for effects from the detector response as a function of the jet $p_{\textrm{T}}$ and $\eta .$ Furthermore, contamination from pileup and electronic noise is subtracted using the charged-hadron subtraction method [61]. To achieve a better agreement between data and simulation, an extra $\eta$-dependent smearing is performed on the jet energy in simulated events [67, 68]. Events are required to have at least two (one) jets with $|\eta |<2.4$ and $p_\textrm{T}>15\,(20)$ $\,\text {Ge}\hspace{-.08em}\text {V}$ in the $\upmu \upmu \text{ b } \text{ b }$ ($\uptau \uptau {\text{ b }}{\text{ b }}$) analysis. Jets are required to be separated from any selected electron, muon, or $\uptau _\textrm{h}$ by $\varDelta R > 0.4\,(0.5)$ in the $\upmu \upmu \text{ b } \text{ b }$ ($\uptau \uptau {\text{ b }}{\text{ b }}$) analysis.

Both channels rely on identifying jets that likely originate from b quarks. The DeepJet flavor classification algorithm [69, 70] is used to tag b jets. Three different working points on the b tagging discriminator values correspond to 0.1, 1, and 10% misidentification probabilities, known respectively as tight (T), medium (M), and loose (L) working points. The misidentification probability to tag a light-flavour jet as a b jet is measured in inclusive QCD multijet MC samples, and they depend on the $p_{\textrm{T}}$ and $\eta$ of the jet. The corresponding b tagging efficiencies, measured in t t+jets events, are about 65, 80, and 95%, respectively [71]. In the $\upmu \upmu \text{ b } \text{ b }$ analysis, the selected jet with the higher b tagging score is required to pass the tight working point whereas the second one fulfills the loose b tagging requirements. In this paper the latter is referred to as the ‘looser’ b jet. In the $\uptau \uptau {\text{ b }}{\text{ b }}$ analysis, the medium working point is used to identify b jets. The shape of the distribution of the b tagging discriminator, and thus the b tagging efficiencies, can be different between data and simulation. Since the $\upmu \upmu \text{ b } \text{ b }$ analysis relies on the b tagging discriminator distribution, shape-based corrections are applied on simulation to match the data. A similar method is used in the $\uptau \uptau {\text{ b }}{\text{ b }}$ final state, which, by construction corrects the b tagging efficiency for all b tagging discriminator scores.

The hadron-plus-strips algorithm [72] with anti-$k_{\textrm{T}}$ jets as seeds is used to reconstruct the hadronically decaying $\uptau$ leptons. The algorithm combines one or three tracks with energy deposits in the calorimeters to identify the $\uptau$ lepton decay modes. Neutral pions are reconstructed as strips with a dynamic size in $\eta$-$\phi$ from reconstructed electrons and photons, where the strip size varies as a function of the $p_{\textrm{T}}$s of the electron or photon candidate. The $p_{\textrm{T}}$ of the $\uptau _\textrm{h}$ candidates are required to be 5$\,\text {Ge}\hspace{-.08em}\text {V}$ greater than the threshold at the trigger level. In events triggered by single leptons, the $\uptau _\textrm{h}$ $p_{\textrm{T}}$ must exceed 20$\,\text {Ge}\hspace{-.08em}\text {V}$. The pseudorapidity of the $\uptau _\textrm{h}$ candidate also depends on the trigger. It is restricted to $|\eta | < 2.1$ if a $\uptau _\textrm{h}$ identification is performed at the HLT, and to $|\eta | < 2.3$ otherwise. To distinguish genuine $\uptau _\textrm{h}$ decays from electrons, muons, or jets originating from the hadronization of quarks or gluons, the DeepTau algorithm [73] is used. Information from all individual reconstructed particles near the $\uptau _\textrm{h}$ candidate axis is combined with properties of the $\uptau _\textrm{h}$ candidate. The probability for a jet to be misidentified as a $\uptau _\textrm{h}$ candidate by the DeepTau algorithm depends on the $p_{\textrm{T}}$ and the jet flavor. In simulated $\text{ W }$+jets events, the misidentification rate for jets is estimated to be 0.4% for a genuine $\uptau _\textrm{h}$ identification efficiency of 70%. The misidentification rate for electrons (muons) is 2.6% (0.03%) for a genuine $\uptau _\textrm{h}$ identification efficiency of 80% ($>99$%). In the $\text{ e } \hspace{-.04em}\uptau _\textrm{h}$ and $\upmu \hspace{-.04em}\uptau _\textrm{h}$ final states of the $\uptau \uptau {\text{ b }}{\text{ b }}$ channel, the $\uptau _\textrm{h}$ candidate must be separated from the electron or muon by $\varDelta R \ge 0.4$ and they must be oppositely charged.

The missing transverse momentum vector ${\vec {p}}_{\textrm{T}}^{\text {miss}}$ is computed as the negative vector sum of the transverse momenta of all the PF candidates in an event, and its magnitude is denoted as $p_{\textrm{T}} ^\text {miss}$  [74]. The ${\vec {p}}_{\textrm{T}}^{\text {miss}}$ is modified to account for corrections to the energy scale of the reconstructed jets in the event. Anomalous high-$p_{\textrm{T}} ^\text {miss}$ events can be due to a variety of reconstruction failures, detector malfunctions or noncollision backgrounds. Such events are rejected by event filters that are designed to identify more than 85–90% of the spurious high-$p_{\textrm{T}} ^\text {miss}$ events with a mistagging rate less than 0.1% [74].

## Event selection

Table 1 summarizes the different $p_{\textrm{T}}$ criteria for online reconstructed electrons, muons and $\uptau _\textrm{h}$s in the $\upmu \upmu \text{ b } \text{ b }$ and $\uptau \uptau {\text{ b }}{\text{ b }}$ channels.

**Fig. 1:**
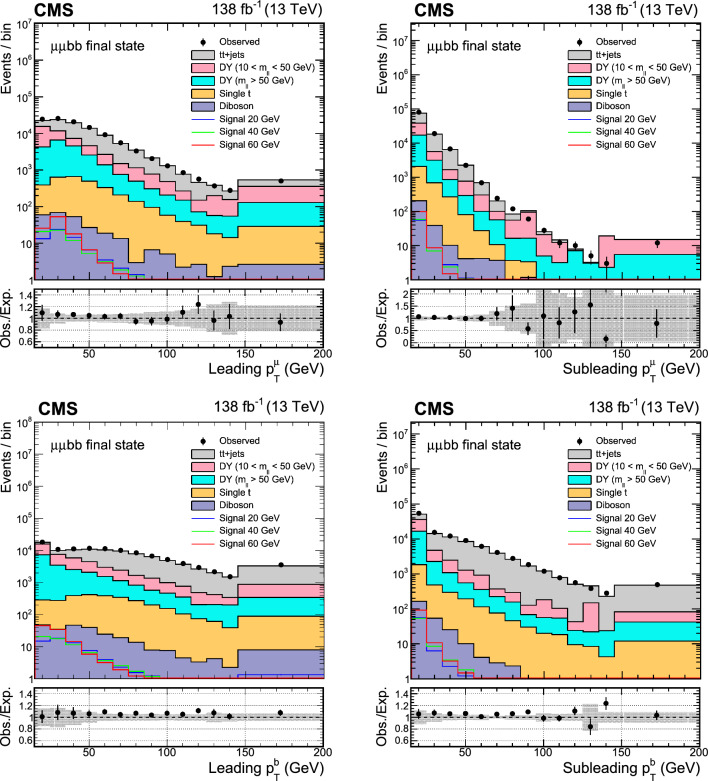
The distributions of leading and subleading (upper) muon $p_{\textrm{T}}$ and (lower) b jet $p_{\textrm{T}}$ in the selected events. The uncertainty band in the lower panel represents the limited size of the simulated samples together with a 30% uncertainty in the low-mass DY cross section. Simulated samples are normalized using the corresponding theoretical cross sections. To evaluate the normalization of the signal, SM Higgs boson cross sections are multiplied by the ${\mathcal {B}}(\text {a}_{1} \text {a}_{1} \rightarrow \upmu \upmu \text{ b } \text{ b } )$ value that is calculated in the Type III model with $\tan \beta = 2$

**Fig. 2:**
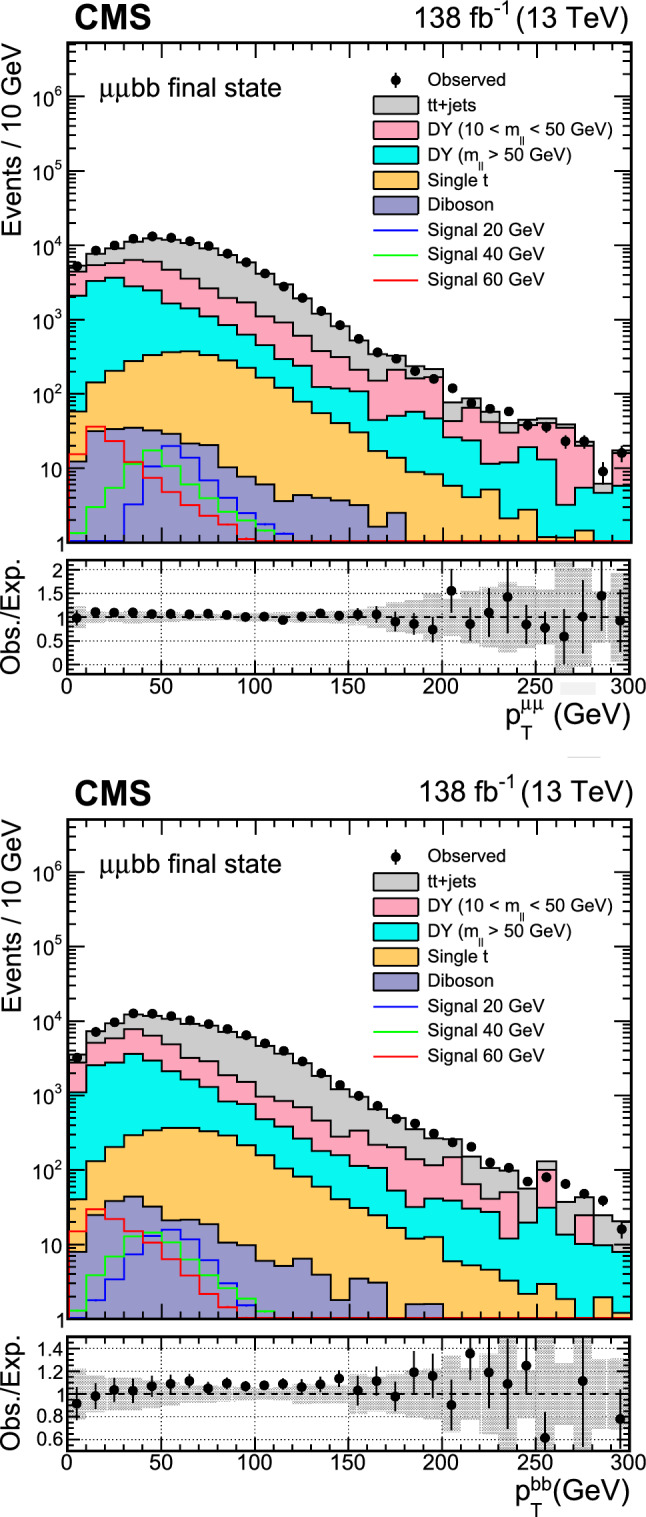
The $p_{\textrm{T}}$ distributions of the (upper) dimuon systems and (lower) di-b-jet system. The uncertainty band in the lower panel represents the limited size of the simulated samples together with a 30% uncertainty in the low-mass DY cross section. Simulated samples are normalized to using the corresponding theoretical cross sections. To evaluate the normalization of the signal, SM Higgs boson cross sections are multiplied by the ${\mathcal {B}}(\text {a}_{1} \text {a}_{1} \rightarrow \upmu \upmu \text{ b } \text{ b } )$ value that is calculated in the Type III model with $\tan \beta = 2$

The $\upmu \upmu \text{ b } \text{ b }$ event candidates are selected based on the requirement that either one or both muons are reconstructed at the HLT. Passing the double-muon trigger necessitates two isolated muons with $p_{\textrm{T}}$ exceeding thresholds of 17 and 8$\,\text {Ge}\hspace{-.08em}\text {V}$, which increases to 24$\,\text {Ge}\hspace{-.08em}\text {V}$ for an isolated muon in the single-muon trigger path. Accepting events from both single- and double-muon triggers improves the trigger efficiency by including events in which the second muon is not reconstructed at the trigger level.

Depending on the decay of the $\uptau$ lepton and the data-taking period, the $\uptau \uptau {\text{ b }}{\text{ b }}$ candidates must pass either a single-electron, single-muon, $\text{ e } \upmu ,$ $\text{ e } \uptau _\textrm{h},$ or $\upmu \uptau _\textrm{h}$ trigger selection. The single-muon, $\text{ e } \upmu$ and $\upmu \uptau _\textrm{h}$ triggers require the reconstructed muon to be isolated, while electron isolation is required for the single-electron, $\text{ e } \upmu$ and $\text{ e } \uptau _\textrm{h}$ triggers. Two $\text{ e } \upmu$ dilepton triggers have been used for all data-taking years, having $p_{\textrm{T}}$ thresholds of 23 (23) and 12 (8)$\,\text {Ge}\hspace{-.08em}\text {V}$ for the $p_{\textrm{T}}$-leading and -subleading lepton of the trigger in the case of electrons (muons). The single-muon and single-electron triggers with $p_{\textrm{T}}$ thresholds of 22 and 25$\,\text {Ge}\hspace{-.08em}\text {V}$, respectively, are used for analyzing the 2016 data. The $p_{\textrm{T}}$ thresholds of electron and $\uptau _\textrm{h}$ candidates are, respectively, 24 and 30$\,\text {Ge}\hspace{-.08em}\text {V}$ for the $\text{ e } \uptau _\textrm{h}$ dilepton trigger in the 2017–2018 data. For the $\upmu \uptau _\textrm{h}$ dilepton trigger, the $p_{\textrm{T}}$ thresholds of muon and $\uptau _\textrm{h}$ are, respectively, 19 (20) and 20 (27)$\,\text {Ge}\hspace{-.08em}\text {V}$ for the data taken during 2016 (2017–2018). The increase in the $p_{\textrm{T}}$ threshold is necessary to control the trigger rate at a larger instantaneous luminosity. Similarly, the $p_{\textrm{T}}$ requirements are tightened for single-lepton triggers across the years. This results in two different thresholds for single-electron and muon triggers for 2017–2018.

Offline, in the $\upmu \upmu \text{ b } \text{ b }$ channel events are required to have two muons and at least two b jets passing the kinematic, identification, and isolation criteria detailed in Sect. 4. While the final search in this channel is performed for $m_{\text {a}_{1}}$ between 15 and 62.5$\,\text {Ge}\hspace{-.08em}\text {V}$, events are selected with a dimuon invariant mass, $m_{\upmu \upmu }$, between 14 and 70$\,\text {Ge}\hspace{-.08em}\text {V}$. The additional sidebands in $m_{\upmu \upmu }$ help model the backgrounds at the boundaries. To reduce the background contribution from t t+jets, events with $p_{\textrm{T}} ^\text {miss} > 60\,\text {Ge}\hspace{-.08em}\text {V}$ are rejected. The selection yields a total of 109 821 data events while the corresponding expected yield from simulated backgrounds is $103\,900 \pm 7300.$ The background contribution should be compared with about 80–100 expected signal events, depending on $m_{\text {a}_{1}}$, from both ${\text{ g } \text{ g }}$F and VBF Higgs boson production. The branching fraction ${\mathcal {B}}(\text {a}_{1} \text {a}_{1} \rightarrow \upmu \upmu \text{ b } \text{ b } )$ is evaluated in the Type III model with $\tan \beta =2.$ Figure 1 shows, in data and simulation, the $p_{\textrm{T}}$ distributions of the $p_{\textrm{T}}$-leading and -subleading muons and b jets. Although the estimation of backgrounds in this analysis does not rely on simulation, the observed level of agreement between data and simulation justifies the use of simulated events to optimize the sensitivity. Figure 2 shows distributions for the $p_{\textrm{T}}$ of the dimuon $(p_{\textrm{T}} ^{\upmu \upmu })$ and the di-b-jet systems $(p_{\textrm{T}} ^{\text{ b } \text{ b }}).$

To further suppress backgrounds, a $\chi _\text {tot} ^2$ variable is defined as $\chi _\text {tot} ^2 = \chi _{\text{ b } \text{ b }} ^2 + \chi _{\text {H} } ^2.$ It examines the compatibility of $m_{\upmu \upmu }$ and $m_{\text{ b } \text{ b }}$ with $m_{\text {a}_{1}}$, and of $m_{\upmu \upmu \text{ b } \text{ b }}$ with the Higgs boson mass in signal events. The components of $\chi _\text {tot} ^2$ are defined as

1 $$\begin{aligned} \chi _{\text{ b } \text{ b }} = \frac{(m_{\text{ b } \text{ b }}- m_{\upmu \upmu })}{\sigma _{\text{ b } \text{ b }}} \quad \text {and} \quad \chi _{\text {H} } = \frac{(m_{\upmu \upmu \text{ b } \text{ b }}- 125\,\text {Ge}\hspace{-.08em}\text {V})}{\sigma _{\text {H} }}. \end{aligned}$$

**Fig. 3:**
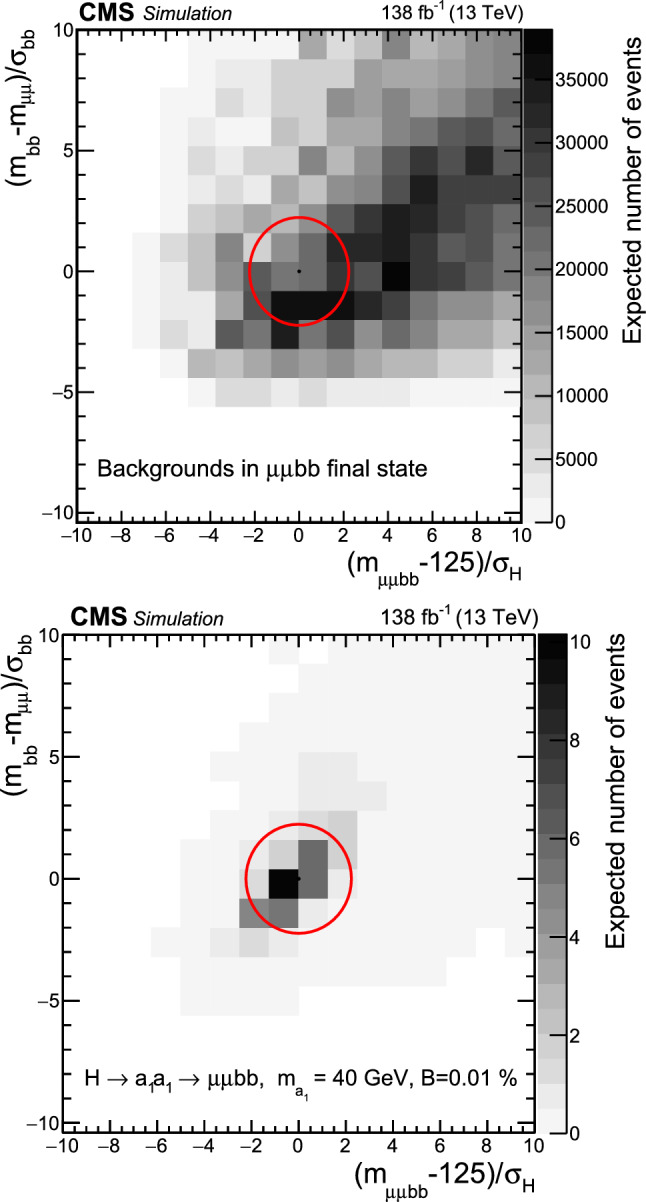
The distribution of $\chi _{\text{ b } \text{ b }}$ versus $\chi _{\text {H} }$ as defined in Eq. (1) for (upper) simulated background processes and (lower) the signal process with $m_{\text {a}_{1}} = 40\,\text {Ge}\hspace{-.08em}\text {V}.$ The contours indicate lines of constant $\chi _\text {tot} ^2.$ The gray scale represents the expected yields in data. To evaluate the yield of the signal, SM Higgs boson cross sections are multiplied by the ${\mathcal {B}}(\text {a}_{1} \text {a}_{1} \rightarrow \upmu \upmu \text{ b } \text{ b } )$ value that is calculated in the Type III model with $\tan \beta =2$

The variables $\sigma _{\text{ b } \text{ b }}$ and $\sigma _{\text {H} }$ are the mass resolutions of the di-b-jet system and the Higgs boson candidate, respectively, which are derived from Gaussian fits to simulated distributions of $m_{\text{ b } \text{ b }}$ and the mass of the Higgs boson candidate. While $\sigma _{\text {H} }$ is found to be constant, $\sigma _{\text{ b } \text{ b }}$ increases linearly with $m_{\text {a}_{1}}$ and is modeled as a function of $m_{\upmu \upmu }$ $(\sigma _{\text{ b } \text{ b }} = am_{\upmu \upmu } + b),$ assuming $m_{\upmu \upmu } =m_{\text {a}_{1}}.$ The $\chi _\text {tot} ^2$ variable is evaluated on an event-by-event basis. It was shown in Ref. [25] that applying a threshold on $\chi _\text {tot} ^2$ leads to a large suppression of backgrounds while keeping the majority of signal events. Such a requirement translates to a circle centered at zero in the 2D-plane of $\chi _{\text{ b } \text{ b }}$ and $\chi _{\text {H} }$, as shown in Fig. 3. However, the $\chi _{\text{ b } \text{ b }}$ and $\chi _{\text {H} }$ components are clearly correlated as can be seen in Fig. 3 (lower). This leads to a loss of signal efficiency when imposing the circular requirement. In addition, both $\chi _{\text{ b } \text{ b }}$ and $\chi _{\text {H} }$ distributions are slightly biased away from zero, adding more inefficiencies. Therefore, in the current analysis the definitions of the variables were adjusted to be unbiased and uncorrelated.

**Fig. 4:**
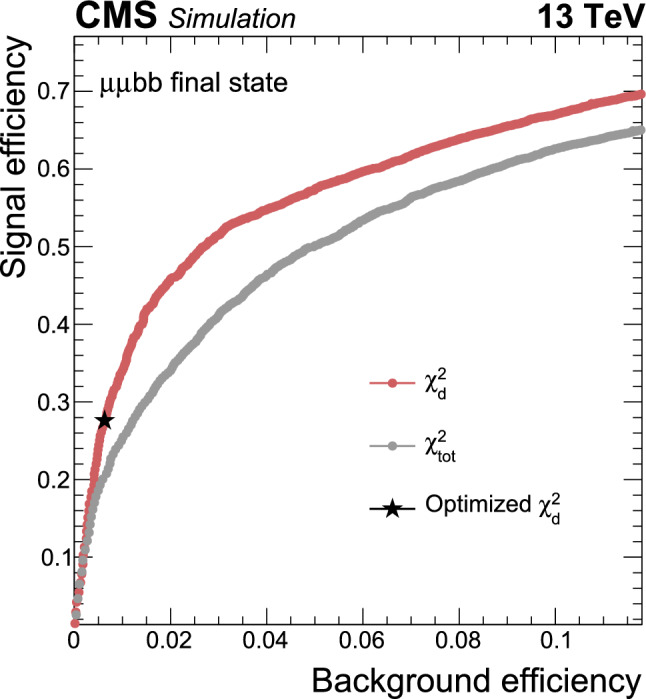
Signal $(m_{\text {a}_{1}} =40\,\text {Ge}\hspace{-.08em}\text {V})$ versus background efficiency for different thresholds on $\chi _\text {tot} ^2$ (gray) and $\chi _{\textrm{d}} ^2$ (red) variables. The black star indicates signal efficiency versus that of background for the optimized $\chi _{\textrm{d}} ^2$ requirement

The correlation between the $\chi _\text {tot}$ components, as well as their bias, depends on $m_{\text {a}_{1}}$. The bias is modeled as a function of $m_{\upmu \upmu }$ and is corrected event by event. After applying this correction, a principal component analysis [75] is performed on the bias-corrected variables. The bias-corrected variables are therefore transformed using the eigenvalues, $\lambda _1$ and $\lambda _2,$ and eigenvectors $$\begin{pmatrix} a\\ b \end{pmatrix},$$ of the correlation matrix:

2 $$\begin{aligned}{} & {} \begin{pmatrix} \chi _{\text {H} } \\ \chi _{\text{ b } \text{ b }} \end{pmatrix}_{\textrm{d}} = \begin{pmatrix} \frac{a}{\sqrt{\lambda _1}}&{}\frac{b}{\sqrt{\lambda _1}}\\ \frac{-b}{\sqrt{\lambda _2}}&{}\frac{a}{\sqrt{\lambda _2}} \end{pmatrix} \begin{pmatrix} \chi _{\text {H} } \\ \chi _{\text{ b } \text{ b }} \end{pmatrix}_{\textrm{c}},\nonumber \\{} & {} \chi _{\textrm{d}} ^2 \equiv \chi _{\text {H} ,{\textrm{d}}} ^2 + \chi _{\text{ b } \text{ b },{\textrm{d}}} ^2 \end{aligned}$$

with subscripts ${\textrm{d}}$ and ${\textrm{c}},$ respectively, standing for decorrelated and bias-corrected components of $\chi _\text {tot}$. The transformation matrix in Eq. (2) has three independent parameters, $a/\sqrt{\lambda _1},$ $a/\sqrt{\lambda _2},$ and *b*/*a*,  that are modeled as functions of $m_{\text {a}_{1}}$. Figure 4 compares the performance of the selection based on $\chi _{\textrm{d}} ^2$ and $\chi _\text {tot} ^2$ variables in terms of the signal $(m_{\text {a}_{1}} =40\,\text {Ge}\hspace{-.08em}\text {V})$ efficiency and background rejection probability. Based on the optimization studies, events with $\chi _{\textrm{d}} ^2<1.5$ are selected.

Table 2 summarizes the number of observed events in data together with the expected yields for the main backgrounds and the signal for different $m_{\text {a}_{1}}$ hypotheses.

**Table 2:** Event yields in the $\upmu \upmu \text{ b } \text{ b }$ channel for simulated processes and the number of observed events in data after applying $\chi _{\textrm{d}} ^2<1.5.$ The expected number of simulated events is normalized to the integrated luminosity of $138{\,\text {fb}^{-1}} .$ The Type III parametrization of 2HDM+S with $\tan \beta =2$ is used to evaluate ${\mathcal {B}}(\text {a}_{1} \text {a}_{1} \rightarrow \upmu \upmu \text{ b } \text{ b } )$

Process	Yield	
t t+jets	$86.3 \pm 2.2$	
DY ($10< m_{\ell \ell } < 50\,\text {Ge}\hspace{-.08em}\text {V}$)	$289.6 \pm 89.5$	
DY ($m_{\ell \ell } > 50\,\text {Ge}\hspace{-.08em}\text {V}$)	$200.2 \pm 31.9$	
Diboson	$1.5 \pm 0.9$	
Single top	$11.4 \pm 1.6$	
Total expected background	$589.1 \pm 95.1$	
Data	641	

Signal for ggH ($\upmu \upmu \text{ b } \text{ b }$)		
$m_{\text {a}_{1}} = 20\,\text {Ge}\hspace{-.08em}\text {V}$	$m_{\text {a}_{1}} = 40\,\text {Ge}\hspace{-.08em}\text {V}$	$m_{\text {a}_{1}} = 60\,\text {Ge}\hspace{-.08em}\text {V}$
15.4 ± 0.2	18.7 ± 0.2	40.5 ± 0.3

**Table 3:** Summary of the categorization requirements in the $\upmu \upmu \text{ b } \text{ b }$ channel. Events in these categories contain two muons and two b jets. As stated in the text, L, M, and T stand for the loose, medium, and tight b tagging criteria, respectively

Categories for selected events	
Low $p_{\textrm{T}}$	At least one b jet with $p_{\textrm{T}} <20\,\text {Ge}\hspace{-.08em}\text {V}$
VBF	Two additional jets with $p_{\textrm{T}} >30\,\text {Ge}\hspace{-.08em}\text {V},$
	$|\eta |<4.7,$ and $m_\textrm{jj}>250\,\text {Ge}\hspace{-.08em}\text {V}$
TL	Looser b jet passes L but fails M
TM	Looser b jet passes M but fails T
TT	Looser b jet passes T

**Table 4:** The expected yields for backgrounds and different signal hypotheses in each category of the $\upmu \upmu \text{ b } \text{ b }$ channel

Category	Signal for ${\text{ g } \text{ g } \text {H} }$ ($\upmu \upmu \text{ b } \text{ b }$)			Expected background
	$m_{\text {a}_{1}} = 20\,\text {Ge}\hspace{-.08em}\text {V}$	$m_{\text {a}_{1}} = 40\,\text {Ge}\hspace{-.08em}\text {V}$	$m_{\text {a}_{1}} = 60\,\text {Ge}\hspace{-.08em}\text {V}$	
Low $p_{\textrm{T}}$	$7.4 \pm 0.1$	$7.3 \pm 0.1$	$17 \pm 0.2$	$421 \pm 88$
VBF	$0.2 \pm (<0.1)$	$1.0 \pm (<0.1)$	$1.1 \pm 0.4$	$5 \pm 2$
TL	$2.1 \pm 0.1$	$2.8 \pm 0.1$	$6.7 \pm 0.1$	$109 \pm 30$
TM	$2.7 \pm 0.1$	$3.3 \pm 0.1$	$7.7 \pm 0.1$	$27 \pm 15$
TT	$2.8 \pm 0.1$	$4.2 \pm 0.1$	$8.1 \pm 0.1$	$28 \pm 11$
Total	$15.4 \pm 0.2$	$18.7 \pm 0.2$	$40.5 \pm 0.3$	$589 \pm 95$

Events are further categorized according to the jet $p_{\textrm{T}}$, the b tagging score of the jets, and additional jet activity in the event compatible with the VBF signature. Events containing at least one of the two selected b jets with $p_{\textrm{T}} < 20\,\text {Ge}\hspace{-.08em}\text {V}$ are put in a separate category (Low $p_{\textrm{T}}$). This category brings extra sensitivity to the signals with lower $m_{\text {a}_{1}}$ values and contains about 70% (40%) background (${\text{ g } \text{ g }}$F signal) events. For the VBF category, events must have at least two jets, in addition to b jets, with $p_{\textrm{T}} >30\,\text {Ge}\hspace{-.08em}\text {V},$ $|\eta |<4.7,$ and an invariant mass $m_\textrm{jj}>250\,\text {Ge}\hspace{-.08em}\text {V}.$ About 50% of VBF signal events fall in this category. The remaining events are categorized based on the b tagging score of the looser b jet. Three exclusive categories are defined where the second jet passes the loose but fails the medium (TL), passes the medium but fails the tight (TM), and passes the tight (TT) b tagging working point. This categorization relies on the fact that events with genuine and misidentified b quark jets are distributed differently among those categories. About 20% of backgrounds as well as the ${\text{ g } \text{ g }}$F signal events fall into the TL category. The TM and TT categories almost equally receive 20% of the ${\text{ g } \text{ g }}$F signal and 5% of the background events. Table 3 summarizes the categories of the current analysis, whereas the expected yields in different categories are presented in Table 4.

In the $\uptau \uptau {\text{ b }}{\text{ b }}$ channel, the offline signal event signature constitutes at least one b jet, and depending on the $\uptau$ lepton decay mode, an $\text{ e } \hspace{-.04em}\upmu$, an $\text{ e } \hspace{-.04em}\uptau _\textrm{h}$, or a $\upmu \hspace{-.04em}\uptau _\textrm{h}$ pair. Any event with an additional electron or muon is rejected to reduce the contribution from DY and multilepton processes. The selection and identification requirements for all objects are discussed in Sect. 4.

Each final state is subdivided into two categories based on the presence of exactly one b jet or at least two b jets in the event. Requiring at least two b jets in the event introduces an additional category compared to Ref. [27], capable of reconstructing the full signal hypothesis and bringing further signal-to-background discrimination power. In total there are six event categories, considering the number of b jets and the decay modes of the $\uptau$ leptons. A deep neural network (DNN) with two hidden layers and 40 nodes is used to discriminate signal from background events in each category. The DNNs are trained using simulated events.

Kinematic properties of the decay products are utilized to construct variables that are inputs to the DNN training, such as the $p_{\textrm{T}}$ and transverse mass ($m_{\textrm{T}}$) of the leptons and b jets, $p_{\textrm{T}}$ and $\eta$ of the di-$\uptau$ system, the invariant mass of each system made of a lepton and a b jet, and $\varDelta R$ between various combinations of the identified particles. One of the most important discriminating observables used in the training is the invariant mass of the decay products of the $\uptau$ leptons and the $p_{\textrm{T}}$-leading b jet, denoted by $m_{b\uptau \uptau }.$ The $m_{b\uptau \uptau }$ value is typically smaller for signal than for background events. Similarly, angular separation and other invariant mass variables can be reconstructed with different combinations of the four final-state particles, employing the correlation between the resonance decay products. The $m_{\textrm{T}}$ between an $\text{ e }$ or $\upmu$ and $p_{\textrm{T}} ^\text {miss}$ is one of the discriminating variables and is defined as

3 $$\begin{aligned} m_{\textrm{T}} (\text{ e }/\upmu , p_{\textrm{T}} ^\text {miss}) \equiv \sqrt{2 p_{\textrm{T}} ^{\text{ e }/\upmu }p_{\textrm{T}} ^\text {miss} \left[ 1 - \cos (\varDelta \phi )\right] }, \end{aligned}$$

where $p_{\textrm{T}} ^{\text{ e }/\upmu }$is the transverse momentum of the lepton and $\varDelta \phi$ is the azimuthal angle between the lepton direction and ${\vec {p}}_{\textrm{T}}^{\text {miss}}$. Events from t t+jets and misidentified $\uptau _\textrm{h}$ backgrounds, such as $\text{ W } \hspace{-.04em}$+jets, have larger $p_{\textrm{T}} ^\text {miss}$, thus result in higher $m_{\textrm{T}}$ values.

Another variable useful in the training is $D_{\zeta }$, defined as

4 $$\begin{aligned} D_{\zeta } \equiv p_{\zeta }- 0.85 p_{\zeta } ^{\text {vis}}, \end{aligned}$$

where the bisector of the directions of the visible $\uptau$ decay products transverse to the beam direction is denoted as the $\zeta$ axis. The quantity $p_{\zeta }$ is defined as the component of the ${\vec {p}}_{\textrm{T}}^{\text {miss}}$ along the $\zeta$ axis, and $p_{\zeta } ^{\text {vis}}$ to be the sum of the components of the lepton transverse momentum along the same direction [76]. The $\text{ Z } \rightarrow \uptau \uptau$ background corresponds to large $D_{\zeta }$ values because the $p_{\textrm{T}} ^\text {miss}$ is approximately collinear to the $\uptau \uptau$ system. The t t+jets events tend to have small $D_{\zeta }$ values due to a large $p_{\textrm{T}} ^\text {miss}$ that is not aligned with the $\uptau \uptau$ system. The signal has intermediate $D_{\zeta }$ values because the $p_{\textrm{T}} ^\text {miss}$ is approximately aligned with the $\uptau \uptau$ system, but its magnitude is small.

For events in the category with two or more b jets, a variable can be constructed to measure the difference between the invariant mass of the two b jets and the invariant mass of the $\uptau \uptau$ system ($m_{\uptau \uptau }$):

5 $$\begin{aligned} \varDelta m_{\text {a}_{1}} \equiv (m_{\text{ b } \text{ b }}-m_{\uptau \uptau })/m_{\uptau \uptau }. \end{aligned}$$

This variable is of particular interest since it peaks at zero for signal events. The $m_{\uptau \uptau }$ distribution reconstructed with the SVfit algorithm [77] is used to test the presence of signal, and thus is not directly included as an input to the DNN.

**Fig. 5:**
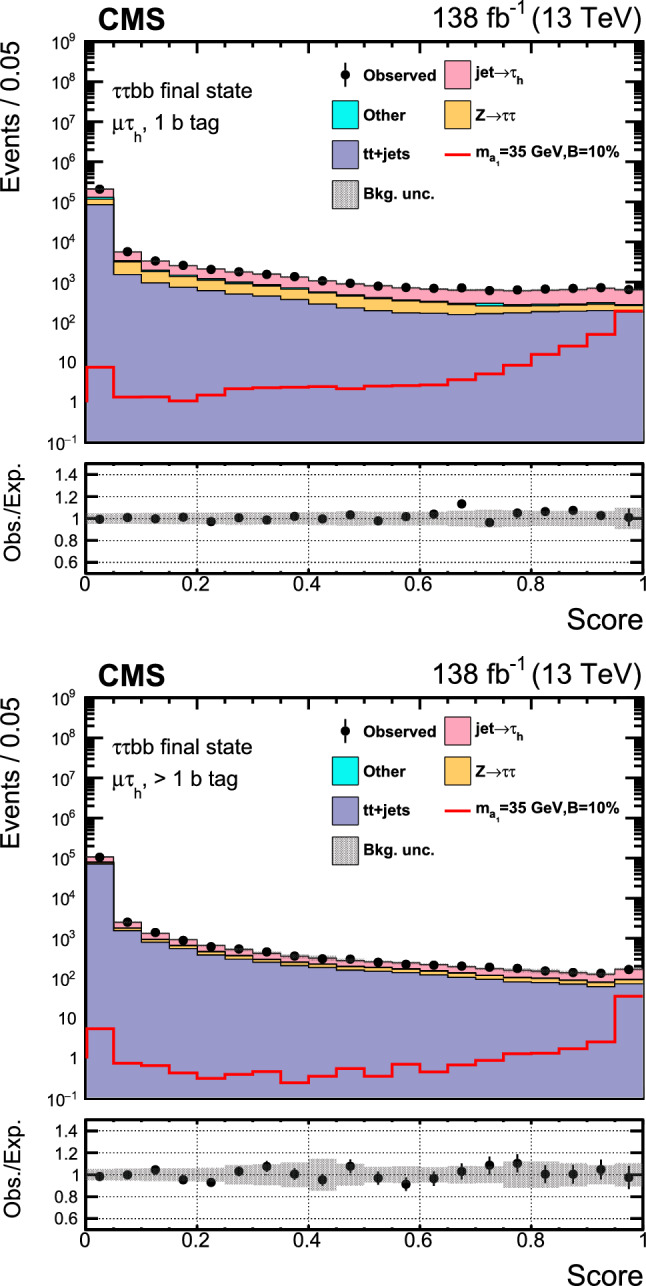
Pre-fit distributions of the DNN score for the $\upmu \hspace{-.04em}\uptau _\textrm{h}$ channel divided into events with one (upper) or at least two (lower) b jets. The shape of the $\text {H} \rightarrow \text {a}_{1} \text {a}_{1}$ signal, where $m_{\text {a}_{1}} = 35\,\text {Ge}\hspace{-.08em}\text {V},$ is indicated assuming ${\mathcal {B}}(\text {H} \rightarrow \text {a}_{1} \text {a}_{1} \rightarrow \uptau \uptau {\text{ b }}{\text{ b }})$ to be 10%. The lower panel shows the ratio of the observed data to the expected yields. The gray band represents the unconstrained statistical and systematic uncertainties

Figure 5 shows, as an example, the DNN score distributions in the $\upmu \hspace{-.04em}\uptau _\textrm{h}$ channel separated for events with one or at least two b jets. The distributions are obtained by comparing the estimated signal and background distributions of the DNN score to that of the data before the fit described in Sect. 7 (pre-fit).

In each category, subregions are defined using a threshold on the DNN score. The expected limits are scanned by varying the DNN thresholds to obtain the highest sensitivity to the simulated signal. This optimization method also ensures that the expected number of background events in each subregion is large enough to perform the final likelihood fit of the $m_{\uptau \uptau }$ distribution. There are three SRs for events containing one b jet: SR1, SR2, and SR3, whereas events with two b jets are divided into two categories: SR1 and SR2. The only exception is the $\text{ e } \hspace{-.04em}\uptau _\textrm{h}$ final state in the two-b-jet category where no significant gain was observed when adding a second signal region. The remaining subregion containing events with the lowest DNN scores is used as a control region (CR) to constrain various background normalizations in the final likelihood fit.

## Background estimation

The presence of a $\upmu \upmu \text{ b } \text{ b }$ signal is expected to appear as a peak over the $m_{\upmu \upmu }$ distribution centered at $m_{\text {a}_{1}}$. The background shape and its normalization in this channel are collectively determined from data with no reference to simulation. Different parameterizations of polynomials are used to model the $m_{\upmu \upmu }$ distribution in data of every category, separately. For each group of models, a maximum degree of the polynomial, determined through statistical tests, is imposed. This is to ensure that the data are not overfit. Parameters of every selected model vary within their uncertainties in the final fit to extract the signal strength, defined as the ratio of the observed signal rate to that predicted by the SM. The latter uses the discrete profiling method [78–80] where every functional form of the selected background models is treated as a discrete nuisance parameter. Along with the determination of the signal strength, one of the background models, its parameters, and the corresponding normalization are determined by the fit, as described in Sect. 7.

**Fig. 6:**
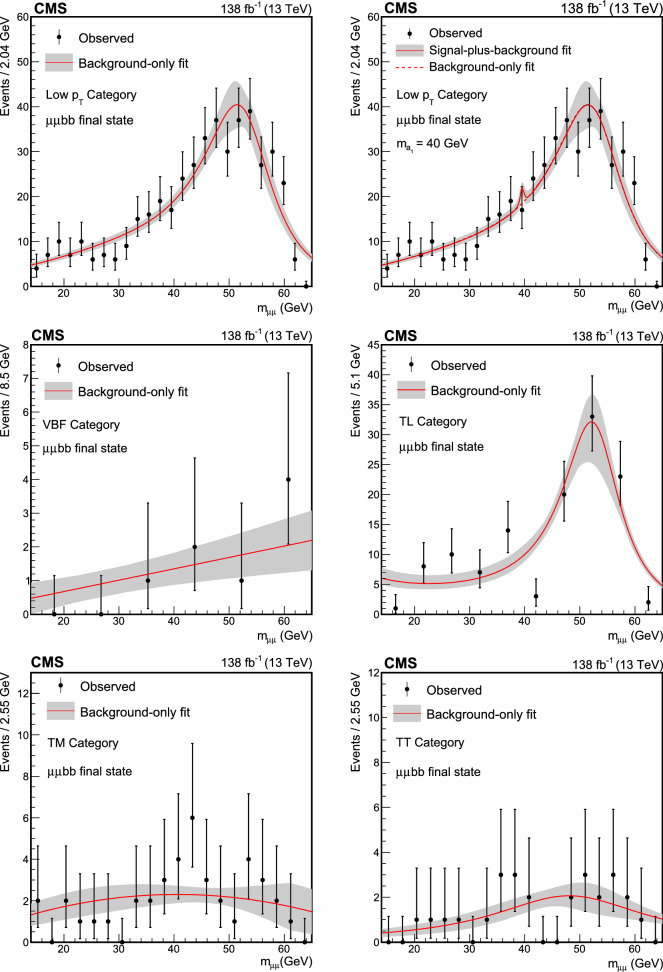
The best fit background models for the $\upmu \upmu \text{ b } \text{ b }$ channel together with a 68% CL uncertainty band from the fit to the data under the background-only hypothesis for the (upper left) Low $p_{\textrm{T}}$ category, (middle left) VBF category, (middle right) TL category, (lower left) TM category, and (lower right) TT category. For comparison, the signal-plus-background is shown for the (upper right) Low $p_{\textrm{T}}$ category for a signal with $m_{\text {a}_{1}} = 40\,\text {Ge}\hspace{-.08em}\text {V}.$ The expected signal yield is evaluated assuming the SM production of the Higgs boson and ${\mathcal {B}}(\text {a}_{1} \text {a}_{1} \rightarrow \upmu \upmu \text{ b } \text{ b } )=0.2\%,$ as predicted in the Type III 2HDM+S with $\tan \beta =2.$ The bin widths depend on statistics, irrelevant for the final fit

A major background contribution to the $\uptau \uptau {\text{ b }}{\text{ b }}$ channel is $\text{ Z } \rightarrow \uptau \uptau ,$ which is estimated from data using an embedding technique [81]. The method is based on the reconstruction of $\text{ Z } \rightarrow \upmu \upmu$ events in data where the muons are replaced with simulated $\uptau$ leptons with the same kinematic properties. In comparison with the simulation of the $\text{ Z } \rightarrow \uptau \uptau$ process, this technique allows a more accurate description of variables related to $p_{\textrm{T}} ^\text {miss}$ and jet activity. The embedded sample also estimates other SM processes with two genuine $\uptau$ leptons, such as t t+jets and Diboson.

The QCD multijet contribution to the $\text{ e } \hspace{-.04em}\upmu$ final state of the $\uptau \uptau {\text{ b }}{\text{ b }}$ channel is estimated using the data in a sideband (SB) region with same-sign $\text{ e } \hspace{-.04em}\upmu$ pairs. The event selection in the SB region is otherwise identical to that in the $\text{ e } \hspace{-.04em}\upmu$ SRs. The contributions of other processes in the SB are taken from simulation and subtracted from the data. The resulting number of data events in the SB is scaled by the ratio of the expected multijet contribution in the SR to the expected multijet contribution in the SB. Scale factors are calculated in data orthogonal to the SR, as functions of the jet multiplicity and the $\varDelta R$ separation between the electron and the muon, in order to account for possible kinematic differences between the two regions.

Backgrounds with hadronic jets that are misidentified as $\uptau _\textrm{h}$ candidates contribute significantly to $\text{ e } \hspace{-.04em}\uptau _\textrm{h}$ and $\upmu \hspace{-.04em}\uptau _\textrm{h}$ final states and are estimated from data. This background includes the $\text{ W } \hspace{-.04em}$+jets, QCD multijets, and t t+jets processes with at least one top quark decaying to hadrons. In a data sideband region, events are required to pass all the baseline $\text{ e } \hspace{-.04em}\uptau _\textrm{h}$/$\upmu \hspace{-.04em}\uptau _\textrm{h}$ selection criteria, but fail the $\uptau _\textrm{h}$ isolation. The data in this SB are reweighted with a factor $f/(1-f),$ where *f* is the probability for a jet to be misidentified as a $\uptau _\textrm{h}$ candidate and is evaluated as a function of the $p_{\textrm{T}} (\uptau _\textrm{h}).$ The $\text{ Z } \rightarrow \upmu \upmu$+jets events in data are used to measure the misidentification probability. The final state must contain a dimuon pair compatible with the decay of the $\text{ Z }$ boson, as well as a $\uptau _\textrm{h}$ candidate. Simulation is used to subtract from data the contribution from events with a genuine $\uptau _\textrm{h}$ lepton. The measurement is done separately for the $\text{ e } \hspace{-.04em}\uptau _\textrm{h}$ and $\upmu \hspace{-.04em}\uptau _\textrm{h}$ final states. This is because the antilepton discrimination working points in the $\uptau _\textrm{h}$ identification change depending on the lepton selected is an electron or a muon [73]. The difference between the two fake rate measurements is observed to be around 10%. The misidentification probability also depends on the jet multiplicity, which characterizes the hadronic activity in the event.

Another dominant background is t t+jets, which has to be carefully estimated from simulation. Because t t+jets events with two genuine $\uptau$ leptons in the final state are an irreducible contribution to the embedded sample described above, the t t+jets background estimate from simulation described here does not include these events. It also does not include t t+jets events in which a reconstructed $\uptau _\textrm{h}$ candidate arises from a simulated jet, as the estimation of the misidentified $\uptau _\textrm{h}$ background is derived from data SBs, as described above. The normalization of backgrounds is free to vary within a range limited by the a priori uncertainty estimates in the final fit for the signal extraction.

The presence of a $\uptau \uptau {\text{ b }}{\text{ b }}$ signal is expected to appear as a peak over the $m_{\uptau \uptau }$ distribution centered at $m_{\text {a}_{1}}$. A fit to the $m_{\uptau \uptau }$ distribution is performed simultaneously in the SRs and CRs described in Sect. 5.

## Signal extraction

In the $\upmu \upmu \text{ b } \text{ b }$ final state, an unbinned maximum likelihood fit to the data $m_{\upmu \upmu }$ distributions is carried out simultaneously in all event categories. The fit is performed in the range $15<m_{\upmu \upmu } <62.5\,\text {Ge}\hspace{-.08em}\text {V},$ using parametric models for signal and background. The parametric model of the signal is a weighted sum of a Voigt profile and a Crystal Ball (CB) function [82], where the mean values of the two are constrained to be identical [25].

Simulated samples are used to determine the parameters of the signal model that may depend on $m_{\text {a}_{1}}$. The studies are performed separately on signal samples simulated for different years. This is to account for the effect of muon reconstruction details on the signal model in different data-taking periods. Most of the parameters are found to be independent of $m_{\text {a}_{1}}$ and fixed in the final fit. Only the resolutions of the Voigt profile and CB function demonstrate linear variation with the pseudoscalar mass. The slope of the linear models are floating parameters in the signal extraction fit. In each category, contributions from different years are normalized considering the signal selection efficiency and acceptance, and are used to construct the expected signal distribution in data. The expected signal efficiency and acceptance are interpolated for $m_{\text {a}_{1}}$ values not covered by simulation.

To evaluate the background contribution, every selected functional form is treated as a discrete nuisance parameter as discussed earlier. In addition, the parameters of every model, as well as the normalization, are part of the background parameter space. A likelihood ${\mathcal {L}}$ is constructed using the signal and the background models in all categories, including systematic uncertainties associated with the signal, as nuisance parameters. In the minimization process of the negative logarithm of the likelihood, the discrete profiling method chooses a best fit background model as the physics parameter of interest, the signal strength, varies. The method incorporates the systematic uncertainty in the background model by taking the envelope of the models provided to the fit.

In practice, a penalty term is added to the likelihood to account for the number of free parameters in the final background model. The penalized likelihood, $\widetilde{{\mathcal {L}}},$ is a function of the measured signal strength, $\mu ,$ the continuous nuisance parameters, $\vec {\theta },$ and the background models, $\vec {b}.$ The penalized likelihood ratio is defined as

6 $$\begin{aligned} -2 \ln \frac{\widetilde{{\mathcal {L}}}({\text {data}}|\mu , \hat{\theta }_\mu , \hat{b}_\mu )}{\widetilde{{\mathcal {L}}}({\text {data}}|\hat{\mu }, \hat{\theta }, \hat{b})}, \end{aligned}$$

with the numerator being the maximum $\widetilde{{\mathcal {L}}}$ for a given $\mu$ at the best fit values of nuisance parameters and background functions. The denominator is the global maximum of $\widetilde{{\mathcal {L}}},$ obtained at $\mu =\hat{\mu },$ $\theta = \hat{\theta },$ and $b = \hat{b}.$ The background function maximizing $\widetilde{{\mathcal {L}}}$ at any $\mu$ is used to derive the confidence interval on $\mu$ at any $m_{\mathrm {a_{1}}}$ [78]. It is verified that the fit is unbiased using studies where signals at several $m_{\text {a}_{1}}$ values are injected with different strengths. The relative change in signal strength is found to be less than $10^{-4}.$ The best fit background models together with their uncertainties are shown in Fig. 6 for all event categories in the $\upmu \upmu \text{ b } \text{ b }$ analysis.

**Fig. 7:**
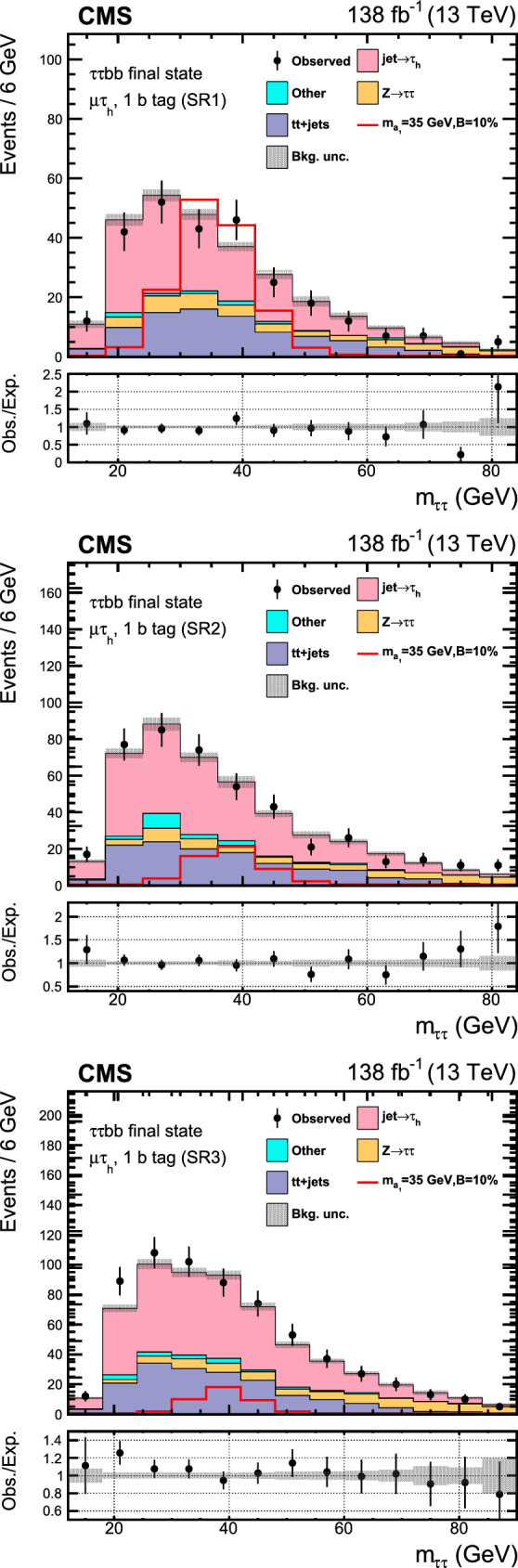
Post-fit distributions of $m_{\uptau \uptau }$ for the $\upmu \hspace{-.04em}\uptau _\textrm{h}$ channel signal regions in events with exactly one b tagged jet: SR1 (upper), SR2 (middle), and SR3 (lower). The shape of the $\text {H} \rightarrow \text {a}_{1} \text {a}_{1}$ signal, where $m_{\text {a}_{1}} = 35\,\text {Ge}\hspace{-.08em}\text {V},$ is indicated assuming ${\mathcal {B}}(\text {H} \rightarrow \text {a}_{1} \text {a}_{1} \rightarrow \uptau \uptau {\text{ b }}{\text{ b }})$ to be 10%

**Fig. 8:**
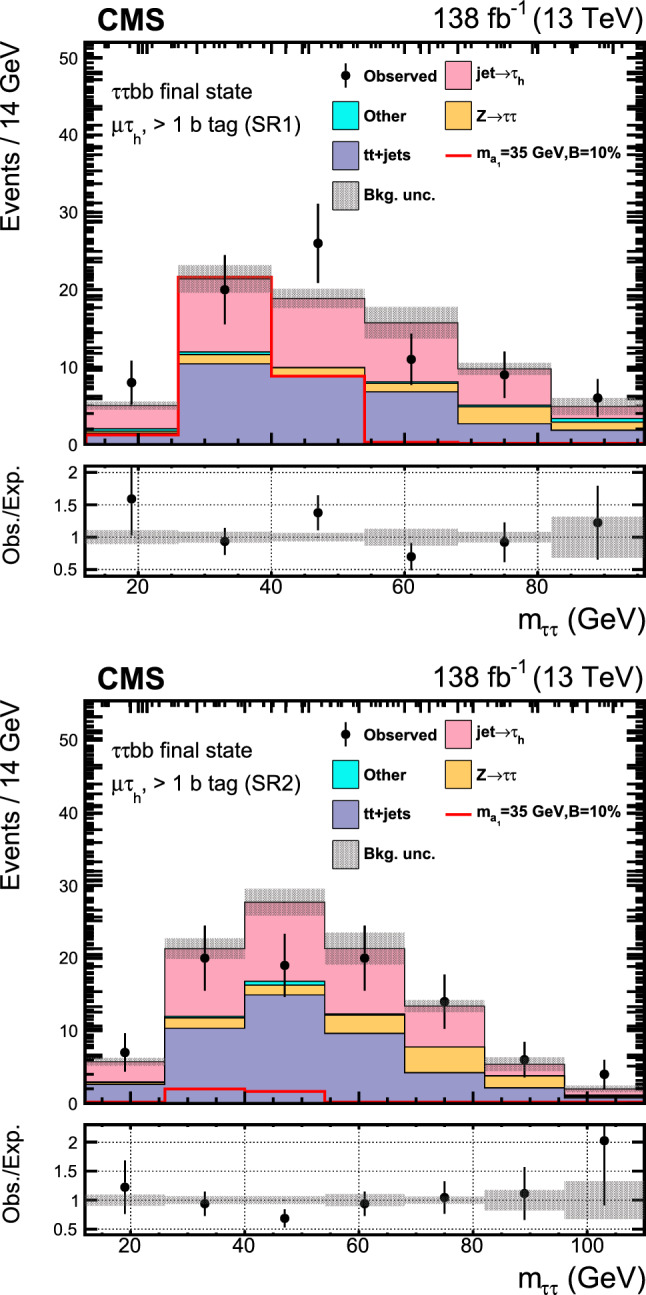
Post-fit distributions of the $m_{\uptau \uptau }$ for the $\upmu \hspace{-.04em}\uptau _\textrm{h}$ channel signal regions in events with at least two b tagged jets: SR1 (upper) and SR2 (lower). The shape of the $\text {H} \rightarrow \text {a}_{1} \text {a}_{1}$ signal, where $m_{\text {a}_{1}} = 35\,\text {Ge}\hspace{-.08em}\text {V},$ is indicated assuming ${\mathcal {B}}(\text {H} \rightarrow \text {a}_{1} \text {a}_{1} \rightarrow \uptau \uptau {\text{ b }}{\text{ b }})$ to be 10%

**Fig. 9:**
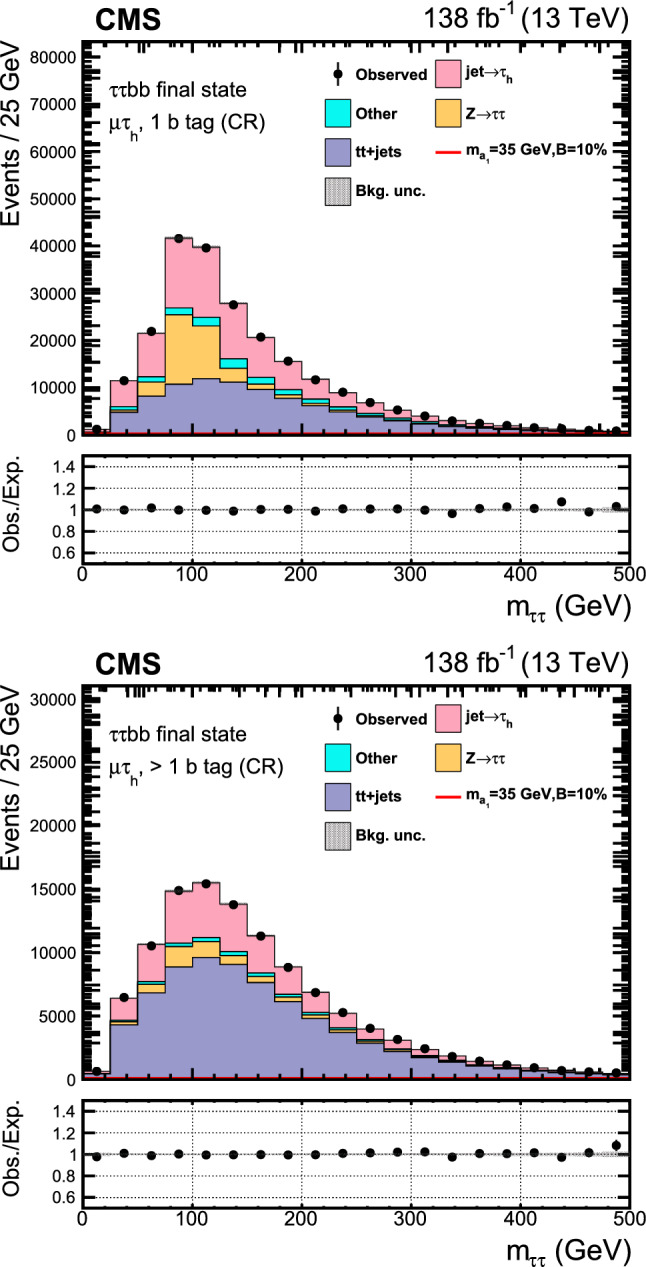
Post-fit distributions of the $m_{\uptau \uptau }$ for the $\upmu \hspace{-.04em}\uptau _\textrm{h}$ channel control regions in events with exactly one b tagged jet (upper) and at least two b tagged jets (lower). The contamination from the $\text {H} \rightarrow \text {a}_{1} \text {a}_{1}$ signal, where $m_{\text {a}_{1}} = 35\,\text {Ge}\hspace{-.08em}\text {V},$ is barely visible assuming ${\mathcal {B}}(\text {H} \rightarrow \text {a}_{1} \text {a}_{1} \rightarrow \uptau \uptau {\text{ b }}{\text{ b }})$ to be 10%

In the $\uptau \uptau {\text{ b }}{\text{ b }}$ channel, a binned maximum likelihood fit is performed on the $m_{\uptau \uptau }$ distribution with systematic uncertainties included as nuisance parameters. The subregions of event categories from all final states are included in a simultaneous fit. Figures 7, 8 and 9 show the post-fit $m_{\uptau \uptau }$ distributions in different subregions and categories for the $\upmu \hspace{-.04em}\uptau _\textrm{h}$ final state.

The limits and confidence intervals are obtained using the modified frequentist $\hbox {CL}_{\textrm{s}}$ approach [83, 84] with an asymptotic approximation to the distribution of the profile likelihood ratio test statistic [85]. Pseudoscalar masses between 12 and 60$\,\text {Ge}\hspace{-.08em}\text {V}$ are considered using simulated samples described in Sect. 3.

The $m_{\upmu \upmu }$ and $m_{\uptau \uptau }$ expected distributions are compared to data in a combined fit, integrating over the $\text {a}_{1}$ decay modes. Integrating over $\text {a}_{1}$ decays makes the combination model dependent since the branching fraction of $\text {a}_{1}$ to fermion pairs depends on the model. The 2HDM+S and the theoretical predictions of Ref. [86] are used for the branching fractions of $\text {a}_{1}$ to muons, $\uptau$ leptons, and b quarks which are fixed in the fit. The selected events are mutually exclusive in the two analyses as events with an extra muon and/or electron are vetoed in the $\uptau \uptau {\text{ b }}{\text{ b }}$ selection. A correlation model is employed between the two analyses for the systematic uncertainties that are in common.

## Systematic uncertainties

The sensitivity of the two analyses, $\upmu \upmu \text{ b } \text{ b }$ and $\uptau \uptau {\text{ b }}{\text{ b }}$, is mainly affected by the uncertainties arising from the finite size of the data sample. Nevertheless, several sources of systematic uncertainties are included in the determination of the results. Most of the systematic uncertainties are common between the two analyses, although their impact on the result may differ. In this class of uncertainties fall those associated with the modeling and acceptance of the signal, including the PDFs, the strong coupling constant, and the renormalization and factorization scales. In addition, experimental uncertainties associated with, e.g., the jet energy calibrations, b tagging, and muon reconstruction and identification are in common between the two analyses, although the uncertainties related to the background estimations are not. In the $\upmu \upmu \text{ b } \text{ b }$ analysis, uncertainties associated with the parameters of the dimuon resonance model in the signal are taken into account.

The unbinned maximum likelihood fit of the $\upmu \upmu \text{ b } \text{ b }$ analysis accounts for the shape uncertainties in a different way. The impact of systematic variations is found to be negligible on the parametric model of the signal for all $m_{\text {a}_{1}}$ hypotheses. On the other hand, the modeling of the $m_{\upmu \upmu }$ resolution with $m_{\text {a}_{1}}$ (discussed in Sect. 7) has an uncertainty that is included in the fit with a Gaussian profile. Uncertainties associated with the background model are evaluated by means of the discrete profiling method as described earlier and contribute to the statistical uncertainty of the result. Depending on the signal mass hypothesis, they constitute about 10–25% of the total uncertainty in the $\upmu \upmu \text{ b } \text{ b }$ results. Contributions from uncertainties in the signal efficiency and acceptance are significantly smaller. In the following, details are provided for several sources of uncertainties.

All uncertainties are included as nuisance parameters in the final fit for the signal extraction. Uncertainties affecting the event yields in categories, i.e., normalization uncertainties, are assigned via multiplicative corrections, with a log-normal probability density function. In the binned maximum likelihood fit of the $\uptau \uptau {\text{ b }}{\text{ b }}$ analysis, nuisance parameters that modify the shapes of the $m_{\uptau \uptau }$ distributions are assumed to have a Gaussian profile. This means that for every nuisance parameter of this type, two alternate distributions are provided to the fit: one with the distribution resulting from an increase of the nuisance parameter by one standard deviation and the other with the distribution resulting from a decrease by one standard deviation. The dominant systematic uncertainty is found to be associated with the signal model, followed by the normalization of the QCD multijet background in the $\text{ e } \hspace{-.04em}\upmu$ final state and the uncertainties in the t t+jets cross section.

**Integrated luminosity**: the integrated luminosity of the data recorded by CMS for physics analyses is evaluated separately for different years of the Run 2 data taking [87–89]. The uncertainty in the measured integrated luminosity of a given year has a component that is uncorrelated across the years. It amounts to 1.0, 2.0, and 1.5%, for the 2016, 2017, and 2018 periods, respectively. Another component is correlated across all three years and is 0.6% in 2016, 0.9% in 2017, and 2.0% in 2018. Furthermore, the luminosity measurements in 2017–2018 have additional uncertainties, of 0.6 and 0.2%, respectively, that are considered correlated between the two years. The overall uncertainty in integrated luminosity for the 2016–2018 period is 1.6%.

**Pileup**: the uncertainty associated with the number of pileup interactions per bunch crossing is estimated by varying the total inelastic pp cross section by 4.6% [90], fully correlated across the years.

**ECAL timing shift**: during the 2016–2017 data-taking periods, a gradual shift in the timing of the ECAL L1 trigger inputs occurred in the forward endcap region, $|\eta | > 2.4$ [91]. This led to a specific inefficiency due to erroneous association of detector readout to the previous bunch crossing in a small fraction of the collision events. A correction to this effect was determined using an unbiased data sample and found to be relevant in events containing high-$p_{\textrm{T}}$ jets with $2.4<|\eta | <3.0.$ This correction is applied to simulation and is accompanied by a 20% uncertainty. The uncertainty predominantly affects the VBF category in the $\upmu \upmu \text{ b } \text{ b }$ analysis, with a negligible effect on the results in this channel.

**Jet energy corrections**: the jet energy scale (JES) uncertainties include several sources parameterized as a function of the jet $p_{\textrm{T}}$ and $\eta$ [92]. Those variations can modify the content of the selected event sample. They also introduce event migration between categories. In the $\upmu \upmu \text{ b } \text{ b }$ analysis, the event $p_{\textrm{T}} ^\text {miss}$ changes as a result of variations in the jet kinematics whereas in the $\uptau \uptau {\text{ b }}{\text{ b }}$ analysis, JES uncertainties affect the $m_{\uptau \uptau }$ distribution. Variations in the expected signal yield are between 15–50% in the $\upmu \upmu \text{ b } \text{ b }$ analysis. In the $\uptau \uptau {\text{ b }}{\text{ b }}$ channel, distributions vary between 10–15% of the nominal. Depending on the source, JES uncertainties are considered as uncorrelated, fully correlated, or partially correlated (50%) across the years. The jet energy resolution is also considered, where the smearing corrections are varied within their uncertainties, uncorrelated across the years.

**b tagging**: sources of systematic uncertainty that affect the data-to-simulation corrections of the b tagging discriminant distribution are JES, the light flavor or gluon (LF) jet contamination in the b jet sample, the heavy flavor (HF) jet contamination in the LF jet sample, and the statistical fluctuations in data and MC [70]. The JES variations in b tagging are obtained together with the JES uncertainties on jet kinematics and follow the same correlation pattern across the years. The statistical components of the b tagging uncertainties are uncorrelated while the rest are assumed correlated between different periods.

**Muon reconstruction**: the data-to-simulation correction factors for the muon tracking, reconstruction and selection efficiencies are estimated using a “tag-and-probe” method [93] in DY data and simulated samples. These uncertainties include the pileup dependence of the correction factors and are correlated across the years since common procedural uncertainties are the dominant source. The requirements between the two analyses are slightly different, mainly because of a different impact parameter in $\uptau \rightarrow \upmu$ decays. The corrections, and therefore associated systematic uncertainties, are applied in bins of muon $p_{\textrm{T}}$ and $|\eta |$ in the $\upmu \upmu \text{ b } \text{ b }$ analysis [63]. In the $\uptau \uptau {\text{ b }}{\text{ b }}$ analysis, a 2% uncertainty, independent of $p_{\textrm{T}}$ and $\eta ,$ per muon is used [94] and treated as uncorrelated between simulated and $\tau$-embedded events. The muon momentum scale varies within 0.4–2.7% [63] and is accounted for in systematic uncertainties on the signal and background $m_{\uptau \uptau }$ distribution. Its impact is found to be negligible in the $\upmu \upmu \text{ b } \text{ b }$ analysis.

**Electron reconstruction**: the electron energy scale uncertainties in $\text{ e } \hspace{-.04em}\upmu$ and $\text{ e } \hspace{-.04em}\uptau _\textrm{h}$ final states are accounted for using methods outlined in Ref. [95]. The reconstruction and selection efficiencies are accompanied by a 2% uncertainty per electron, independent of $p_{\textrm{T}}$ and $\eta$ [64]. Similar to muons, these uncertainties are uncorrelated between simulated and $\tau$-embedded events. Uncertainties in the electron energy scale also affect the shapes of the $m_{\uptau \uptau }$ distributions and are accounted for.

**Fig. 10:**
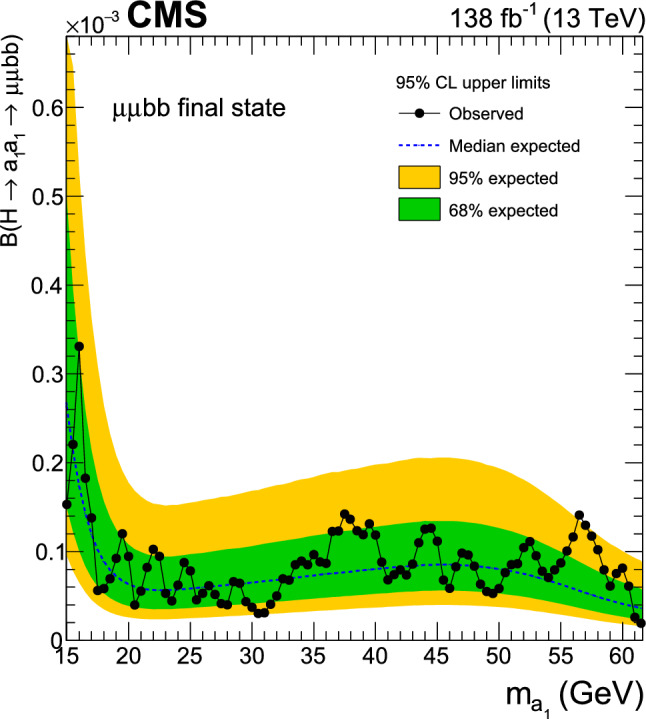
Observed and expected upper limits at 95% $\text {CL}$ on ${\mathcal {B}}(\text {H} \rightarrow \text {a}_{1} \text {a}_{1} \rightarrow \upmu \upmu \text{ b } \text{ b } )$ as functions of $m_{\text {a}_{1}}$. The inner and outer bands indicate the regions containing the distribution of limits located within 68 and 95% confidence intervals, respectively, of the expectation under the background-only hypothesis

**Hadronically decaying** $\uptau$ **lepton reconstruction**: in $\upmu \hspace{-.04em}\uptau _\textrm{h}$ and $\text{ e } \hspace{-.04em}\uptau _\textrm{h}$ final states, there are uncertainties associated with $\uptau _\textrm{h}$ identification efficiencies and energy scale corrections where the variations depend on $p_{\textrm{T}}$ ($\uptau _\textrm{h}$) and decay mode, ranging from 3–5% and 0.2–1.1%, respectively. Systematic variations in the selected event yields as well as in the shapes of the distributions are taken into account. Uncertainties are considered uncorrelated across the bins of $p_{\textrm{T}}$ ($\uptau _\textrm{h}$) and different years for the MC [72]. Uncertainties of the same source are treated as 50% correlated between the embedded DY background and simulated samples. For events with a genuine $\uptau _\textrm{h}$ lepton matched at the generator level, energy scale uncertainties are considered using shape variations. In the case of muons and electrons misidentified as $\uptau _\textrm{h}$ candidates, energy scale corrections are applied in bins of $p_{\textrm{T}}$, $\eta ,$ and decay mode of the misidentified $\uptau _\textrm{h}$. These corrections are associated with uncertainties. A 50% correlation is considered between the embedded and MC samples for these lepton energy scale uncertainties.

**Trigger efficiencies**: an uncertainty of 1% is assigned to the HLT efficiency in the $\upmu \upmu \text{ b } \text{ b }$ analysis. In the $\uptau \uptau {\text{ b }}{\text{ b }}$ channel, an uncertainty of 2% is applied per single-lepton trigger and 5–10% on the dilepton triggers with a $\uptau _\textrm{h}$ requirement. Uncertainties associated with trigger efficiencies affect the shape of the distributions in this channel. The shape effects are taken into account in both simulated and embedded backgrounds, where a 50% correlation is considered between the two.

**Background estimations in** $\mathbf {\uptau \uptau {\text{ b }}{\text{ b }}}$ **final state**: the $\text{ Z }$ boson $p_{\textrm{T}}$ reweighting uncertainty in DY samples, which amounts to 10% of the nominal value, is taken as a $m_{\uptau \uptau }$ shape uncertainty. The embedded samples include a 4% normalization uncertainty [81]. Moreover, shape uncertainties related to tracking efficiencies and contamination from non-DY events in the embedded sample are considered. Since the contribution of the QCD multijet background in the $\text{ e } \hspace{-.04em}\upmu$ channel is obtained from a same-sign sideband region with a limited number of events, the validity of the method is tested in independent same-sign SBs. This test results in a 20% normalization uncertainty. The uncertainty in the scale factor between the same-sign SBs and opposite-sign SRs is modeled using shape variations in the fit used to obtain the nominal values. The misidentification probability, *f*,  of a jet as a $\uptau _\textrm{h}$ candidate depends on the jet multiplicity. A 20% normalization uncertainty is applied to the estimate of the $\text{ W } \hspace{-.04em}$+jets and QCD multijet backgrounds due to *f* being measured in $\text{ Z } \rightarrow \upmu \upmu$ events with different jet multiplicities. In addition, shape variations due to different measurements of *f* are considered.

**Fig. 11:**
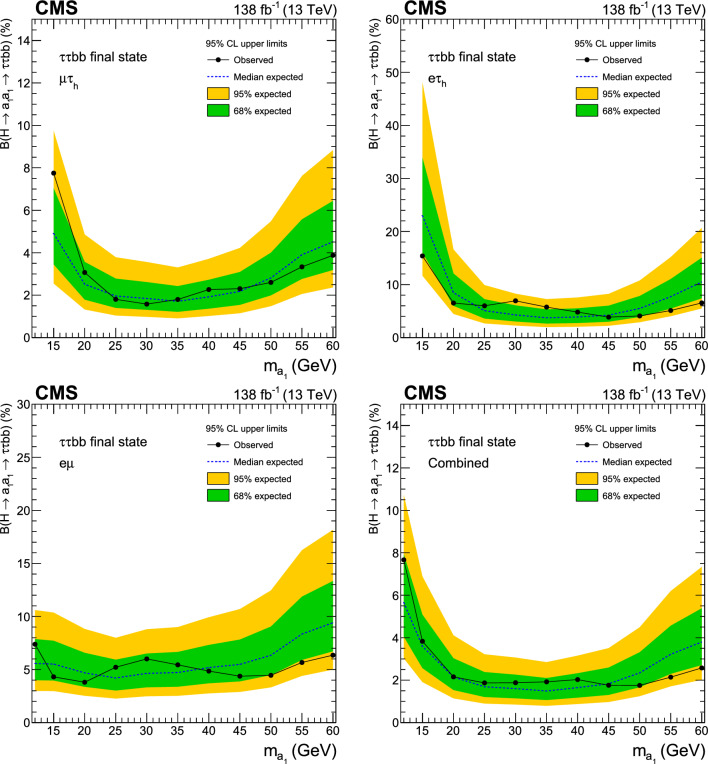
Observed and expected 95% $\text {CL}$ exclusion limits on ${\mathcal {B}}(\text {H} \rightarrow \text {a}_{1} \text {a}_{1} \rightarrow \uptau \uptau {\text{ b }}{\text{ b }})$ in percent, for the (upper left) $\upmu \hspace{-.04em}\uptau _\textrm{h}$, (upper right) $\text{ e } \hspace{-.04em}\uptau _\textrm{h}$, (lower left) $\text{ e } \hspace{-.04em}\upmu$ channels, and (lower right) for the combination of all the channels

**Limited size of the samples**: to account for the limited size of the simulated samples, as well as the data in SBs used to estimate backgrounds, a bin-by-bin statistical uncertainty is considered where a Poisson nuisance parameter per bin is assigned to distributions in those samples [96]. This uncertainty is specific to the $\uptau \uptau {\text{ b }}{\text{ b }}$ analysis.

**Modeling uncertainties**: a total uncertainty of 3.6% is assigned to the sum of the ggF and VBF Higgs boson production cross sections [22] predicted by the SM and used to describe the upper limits on ${\mathcal {B}}(\text {H} \rightarrow \text {a}_{1} \text {a}_{1} \rightarrow \upmu \upmu \text{ b } \text{ b } /\uptau \uptau {\text{ b }}{\text{ b }}).$ It includes uncertainties from the perturbative QCD calculations, PDFs, and $\alpha _\textrm{S}$. In the $\uptau \uptau {\text{ b }}{\text{ b }}$ analysis, PDF and $\alpha _\textrm{S}$ uncertainties are considered for simulated backgrounds, namely: 4.2% for t t+jets, 5% for Diboson, and 5% for single top quark processes. These uncertainties are obtained following the PDF4LHC prescription [97]. To account for variations in the signal acceptance in both channels, the renormalization and factorization scales are doubled and halved simultaneously in simulation. In addition, the eigenvectors of the NNPDF3.1 PDF set are varied within their uncertainties in the final fit. The value of $\alpha _\textrm{S},$ computed at the energy scale of the $\text{ Z }$ boson mass, is also varied within its uncertainty in the PDF set. For the parton shower simulation, uncertainties are separately assessed for initial- and final-state radiation, by varying the respective scales up and down by factors of two. Using the same model assumptions and procedures, the aforementioned uncertainties are considered fully correlated across the data-taking years.

## Results

No excess of events over the expected SM backgrounds is observed in either of the $\upmu \upmu \text{ b } \text{ b }$ and $\uptau \uptau {\text{ b }}{\text{ b }}$ channels. Upper limits are placed, at 95% $\text {CL}$, on ${\mathcal {B}}(\text {H} \rightarrow \text {a}_{1} \text {a}_{1} \rightarrow \ell \ell \text{ b } \text{ b})$ as a function of $m_{\text {a}_{1}}$, with $\ell$ being either a $\uptau$ lepton or muon. The two final states are combined to set upper limits on ${\mathcal {B}}(\text {H} \rightarrow \text {a}_{1} \text {a}_{1} ),$ assuming fixed decay fractions of $\text {a}_{1}$. The branching fraction ${\mathcal {B}}(\text {a}_{1} \rightarrow {\textrm{ff}})$ depends on the 2HDM+S parameters, where f indicates either muon, b quark, or $\uptau$ lepton. Since the results in both channels are statistically limited, the combination mostly benefits from the additional data. The combined results are still dominated by the statistical uncertainties. At $m_{\text {a}_{1}} =35\,\text {Ge}\hspace{-.08em}\text {V},$ all systematic uncertainties amount to about 6% of the total uncertainty, with the dominant contributions corresponding to JES in the $\upmu \upmu \text{ b } \text{ b }$ channel, followed by the theoretical uncertainties in the signal, and finally the uncertainties in the QCD multijet backgrounds in the $\text{ e } \hspace{-.04em}\upmu$ final state of the $\uptau \uptau {\text{ b }}{\text{ b }}$ analysis.

Figure 10 shows the upper limits on ${\mathcal {B}}(\text {H} \rightarrow \text {a}_{1} \text {a}_{1} \rightarrow \upmu \upmu \text{ b } \text{ b } )$ at 95% $\text {CL}$, assuming SM predictions for the Higgs boson production cross section. The $\upmu \upmu \text{ b } \text{ b }$ search is optimized for $m_{\text {a}_{1}}$ values between 15 and 60$\,\text {Ge}\hspace{-.08em}\text {V}$, with signal sensitivity falling rapidly below $m_{\text {a}_{1}} =20\,\text {Ge}\hspace{-.08em}\text {V}.$ This is mainly because the two b jets start to merge as a result of a higher momentum for $\text {a}_{1}$. At 95% $\text {CL}$, the observed upper limits are (0.17–3.3) $\times 10^{-4}$ for the mass range 15 to 62.5$\,\text {Ge}\hspace{-.08em}\text {V}$, while the expected limits are (0.35–2.6) $\times 10^{-4}.$

Figure 11 shows the observed and expected 95% $\text {CL}$ upper limits on ${\mathcal {B}}(\text {H} \rightarrow \text {a}_{1} \text {a}_{1} \rightarrow \uptau \uptau {\text{ b }}{\text{ b }})$ as functions of $m_{\text {a}_{1}}$. Only the $\text{ e } \hspace{-.04em}\upmu$ channel provides sensitivity to the 12$\,\text {Ge}\hspace{-.08em}\text {V}$ mass point, as in this channel the baseline selection on the $\varDelta R$ between the two $\uptau$ candidates is the lowest. For small $m_{\text {a}_{1}}$ values, the decay products appear as boosted and may not be reconstructed as two separate objects. The low $\varDelta R$ requirement allows a selection of more signal events where the two $\uptau$ candidates are close to each other. The $\upmu \hspace{-.04em}\uptau _\textrm{h}$ final state is the most sensitive, where limits as low as around 1.8% (1.7%) are observed (expected) in the intermediate mass range at $m_{\text {a}_{1}} = 35\,\text {Ge}\hspace{-.08em}\text {V}.$ Combining all final states in the $\uptau \uptau {\text{ b }}{\text{ b }}$ channel, observed limits on the branching fraction are found to be in the range 1.7–7.7%, for a pseudoscalar mass between 12 and 60$\,\text {Ge}\hspace{-.08em}\text {V}$, with corresponding expected limits in the range 1.5–5.7% at 95% $\text {CL}$.

**Fig. 12:**
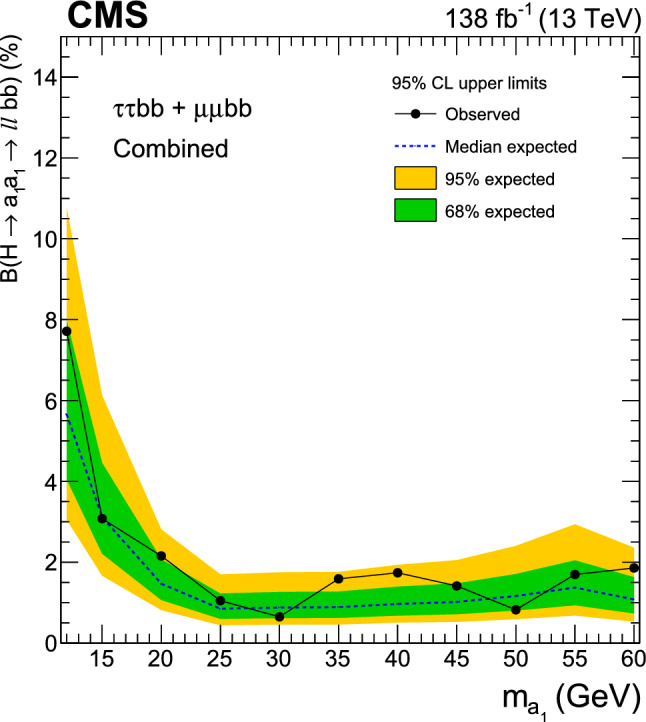
Observed and expected 95% CL upper limits on ${\mathcal {B}}(\text {H} \rightarrow \text {a}_{1} \text {a}_{1} \rightarrow \ell \ell \text{ b } \text{ b})$ in %, where $\ell$ stands for muons or $\uptau$ leptons, obtained from the combination of the $\upmu \upmu \text{ b } \text{ b }$ and $\uptau \uptau {\text{ b }}{\text{ b }}$ channels. The results are obtained as functions of $m_{\text {a}_{1}}$ for 2HDM+S models, independent of the type and $\tan \beta$ parameter

Figure 12 shows the observed and expected limits at 95% $\text {CL}$ on ${\mathcal {B}}(\text {H} \rightarrow \text {a}_{1} \text {a}_{1} \rightarrow \ell \ell \text{ b } \text{ b}),$ where $\ell$ stands for muons or $\uptau$ leptons. Using decay width expression from Ref. [86], the signal strength of each channel is scaled with a type and $\tan \beta$ independent factor to obtain this limit in the context of 2HDM+S models. The observed and expected ranges are 0.6–7.7% and 0.8–5.7% respectively, depending on $m_{\text {a}_{1}}$.

**Fig. 13:**
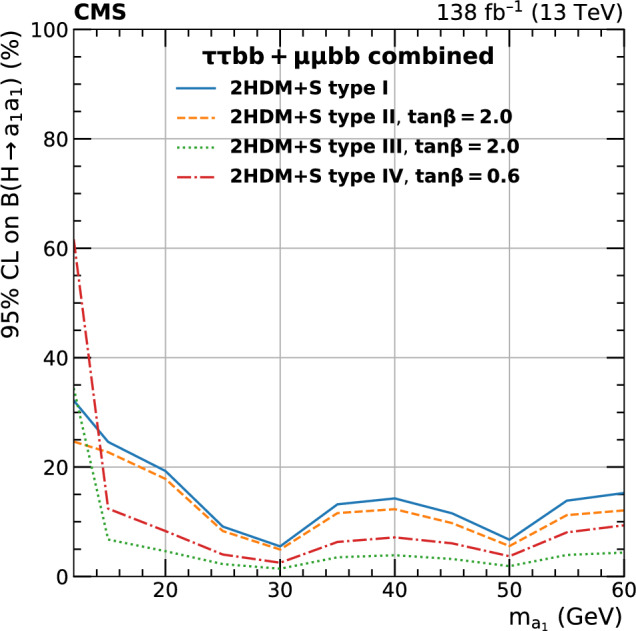
Observed and expected 95% CL upper limits on ${\mathcal {B}}(\text {H} \rightarrow \text {a}_{1} \text {a}_{1} )$ in %, obtained from the combination of the $\upmu \upmu \text{ b } \text{ b }$ and $\uptau \uptau {\text{ b }}{\text{ b }}$ channels. The results are obtained as functions of $m_{\text {a}_{1}}$ for 2HDM+S Type I (independent of $\tan \beta$), Type II $(\tan \beta =2.0),$ Type III $(\tan \beta =2.0),$ and Type IV $(\tan \beta =0.6),$ respectively

The combined branching fraction ${\mathcal {B}}(\text {H} \rightarrow \text {a}_{1} \text {a}_{1} )$ is obtained upon reinterpretation of the $\upmu \upmu \text{ b } \text{ b }$ and $\uptau \uptau {\text{ b }}{\text{ b }}$ results in different types of 2HDM+S and $\tan \beta$ values for $15<m_{\text {a}_{1}} <60\,\text {Ge}\hspace{-.08em}\text {V},$ illustrated in Fig. 13. Upper limits in the range 5–23% are observed at 95% $\text {CL}$ for all Type II scenarios with $\tan \beta > 1.0.$ The tightest constraint is obtained for the Type III scenario with $\tan \beta = 2.0.$ At 95% $\text {CL}$, the observed upper limits on the combined branching fraction are in the range 1–7%, with a similar range for the expected upper limits. For the Type IV scenario, the observed upper limits on ${\mathcal {B}}(\text {H} \rightarrow \text {a}_{1} \text {a}_{1} )$ at 95% $\text {CL}$ are between about 3 and 15% for $\tan \beta = 0.5,$ with corresponding expected limits between about 3 and 11%.

**Fig. 14:**
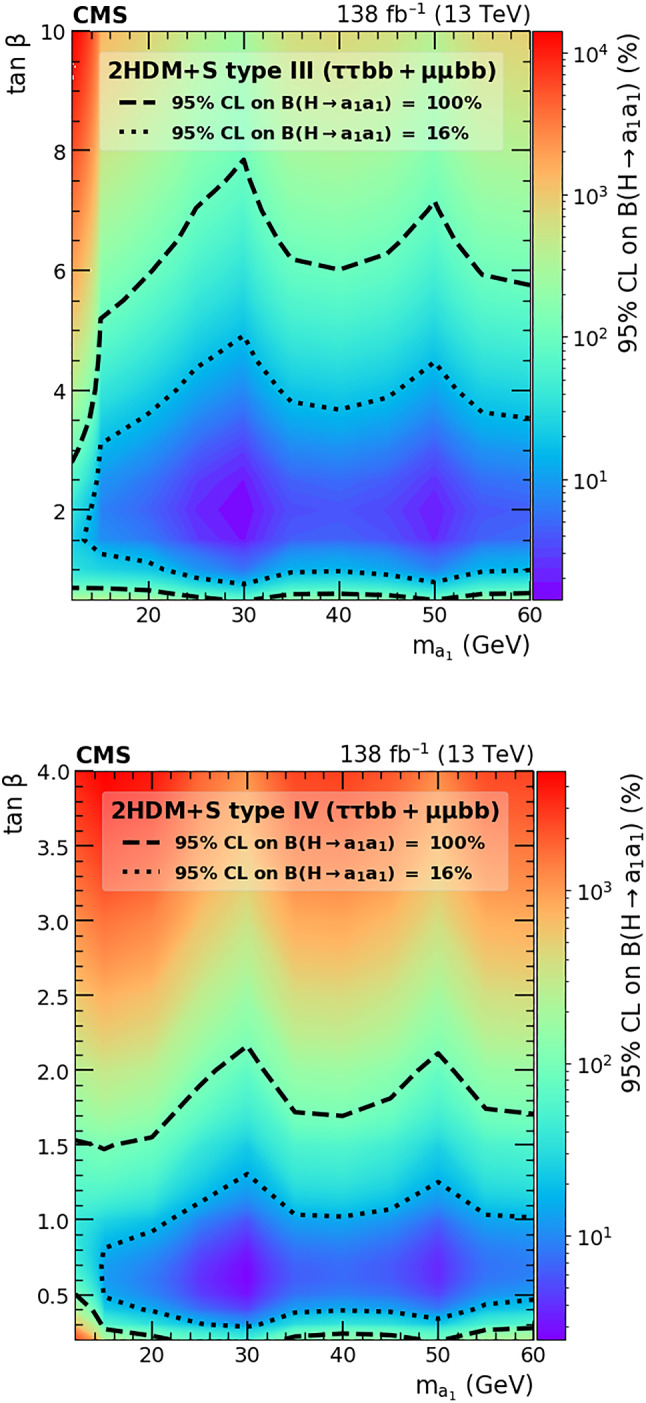
Observed 95% CL upper limits on ${\mathcal {B}}(\text {H} \rightarrow \text {a}_{1} \text {a}_{1} )$ in %, for the combination of the $\upmu \upmu \text{ b } \text{ b }$ and $\uptau \uptau {\text{ b }}{\text{ b }}$ channels for Type III (upper) and Type IV (lower) 2HDM+S in the $\tan \beta$ *vs.* $m_{\text {a}_{1}}$ parameter space. The limits are calculated in a grid of 5$\,\text {Ge}\hspace{-.08em}\text {V}$ in $m_{\text {a}_{1}}$ and 0.1–0.5 in $\tan \beta$, interpolating the points in between. The contours corresponding to branching fractions of 100 and 16% are drawn using dashed lines, where 16% refers to the combined upper limit on Higgs boson to undetected particle decays from previous Run 2 results [16]. All points inside the contour are allowed within that upper limit

The allowed values of $\tan \beta$ and $m_{\text {a}_{1}}$ are shown in Fig. 14 in the context of Type III and Type IV 2HDM+S. The dashed contours represent the upper limits at 95% $\text {CL}$ on Higgs boson to pseudoscalar decays, assuming the branching fraction to be either 100 or 16%. Here 16% corresponds to the combined upper limit on Higgs boson to BSM particle decays obtained from previous Run 2 results [16].

## Summary

A search for an exotic decay of the 125$\,\text {Ge}\hspace{-.08em}\text {V}$ Higgs boson ($\text {H}$) to a pair of light pseudoscalar bosons ($\text {a}_{1}$) in the final state with two b quarks and two muons or two $\uptau$ leptons has been presented. The results are based on a data sample of proton–proton collisions corresponding to an integrated luminosity of 138$\,\text {fb}^{-1}$ , accumulated by the CMS experiment at the LHC during Run 2 at a center-of-mass energy of 13$\,\text {Te}\hspace{-.08em}\text {V}$. Final states with at least one leptonic $\uptau$ decay are studied in the $\uptau \uptau {\text{ b }}{\text{ b }}$ channel, excluding those with two muons or two electrons. The results show significant improvement, with respect to the earlier CMS analyses at 13$\,\text {Te}\hspace{-.08em}\text {V}$, beyond what is merely expected from the increase in the size of the data sample. A more thorough analysis of the signal properties using a single discriminating variable improves the $\upmu \upmu \text{ b } \text{ b }$ analysis, while the $\uptau \uptau {\text{ b }}{\text{ b }}$ analysis gains from a deep neural network based signal categorization. No significant excess in the data over the standard model backgrounds is observed. Upper limits are set, at 95% confidence level, on branching fractions ${\mathcal {B}}(\text {H} \rightarrow \text {a}_{1} \text {a}_{1} \rightarrow \upmu \upmu \text{ b } \text{ b } )$ and ${\mathcal {B}}(\text {H} \rightarrow \text {a}_{1} \text {a}_{1} \rightarrow \uptau \uptau {\text{ b }}{\text{ b }}),$ in the $\upmu \upmu \text{ b } \text{ b }$ and $\uptau \uptau {\text{ b }}{\text{ b }}$ analyses, respectively. Both analyses provide the most stringent expected limits to date. In the $\upmu \upmu \text{ b } \text{ b }$ channel, the observed limits are in the range (0.17–3.3) $\times 10^{-4}$ for a pseudoscalar mass, $m_{\text {a}_{1}}$, between 15 and 62.5$\,\text {Ge}\hspace{-.08em}\text {V}$. Combining all final states in the $\uptau \uptau {\text{ b }}{\text{ b }}$ channel, limits are observed to be in the range 1.7–7.7% for $m_{\text {a}_{1}}$ between 12 and 60$\,\text {Ge}\hspace{-.08em}\text {V}$. By combining the $\upmu \upmu \text{ b } \text{ b }$ and $\uptau \uptau {\text{ b }}{\text{ b }}$ channels, upper limits are set on the branching fraction ${\mathcal {B}}(\text {H} \rightarrow \text {a}_{1} \text {a}_{1} \rightarrow \ell \ell \text{ b } \text{ b}),$ where $\ell$ stands for muons or $\uptau$ leptons. The observed upper limits range between 0.6 and 7.7% depending on the $m_{\text {a}_{1}}$. The results can also be interpreted in different types of 2HDM+S models. For $m_{\text {a}_{1}}$ values between 15 and 60$\,\text {Ge}\hspace{-.08em}\text {V}$, ${\mathcal {B}}(\text {H} \rightarrow \text {a}_{1} \text {a}_{1} )$ values above 23% are excluded, at 95% confidence level, in most of the Type II scenarios. In Types III and IV, observed upper limits as low as 1 and 3% are obtained, respectively, for $\tan \beta =2.0$ and 0.5.

## Data Availability

This manuscript has associated data in a data repository. [Author’s comment: Release and preservation of data used by the CMS Collaboration as the basis for publications is guided by the CMS policy as stated in “https://cms-docdb.cern.ch/cgi-bin/PublicDocDB/RetrieveFile?docid=6032 &filename=CMSDataPolicyV1.2.pdf &version=2. CMS data preservation, re-use and open access policy.]
